# Optimization of photobiomodulation therapy for spinal cord injury: A review

**DOI:** 10.1111/php.70041

**Published:** 2025-11-05

**Authors:** Isabella K. M. Drew, Alan R. Harvey, Vincent P. Wallace, Stuart I. Hodgetts

**Affiliations:** ^1^ School of Human Sciences The University of Western Australia Crawley Western Australia Australia; ^2^ Perron Institute for Neurological and Translational Science Nedlands Western Australia Australia; ^3^ Department of Physics The University of Western Australia Crawley Western Australia Australia

**Keywords:** mechanism, optimization, parameters, pathophysiology, photobiomodulation, spinal cord injury

## Abstract

Interrelated secondary events occur within days and weeks following a spinal cord injury (SCI), constituting a major hurdle in providing both an effective and affordable treatment for spinal cord repair in that it requires a multifaceted approach. Photobiomodulation (PBM) therapy in the red/near‐infrared spectrum holds promising reparative potential; however, there are no consistent or defined parameters for PBM delivery, which may explain the limited number of ongoing clinical trials and less‐than‐optimal reported outcomes. This review outlines the associated complexities of the secondary cascade after SCI, with insights on how and when red/near‐infrared irradiation may alleviate these issues. The primary focus is to discuss limitations within the field that may be inhibiting our ability to characterize optimal guidelines and specifications. Ultimately, this review provides a call for action, as there is an urgent need for consensus and standardization of therapeutic preclinical methodologies if we hope to develop treatment protocols that provide a first‐line minimally invasive therapy to (i) minimize injury sequelae and (ii) facilitate spinal cord repair. We recommend establishing a universal method to measure the therapeutic dose of light delivered to an injury site and employing standardized methodologies across all studies to assess the benefits of PBM therapy.

## Graphical abstract

Photobiomodulation (PBM) therapy in the red and near‐infrared range can significantly modulate the secondary injury response and promote the reparative and regenerative potential of neural tissue after spinal cord injury (SCI). At present, due to the nature of delivery methods, the most effective dose and irradiance at the injury site to optimize therapeutic benefits are unknown. This review focuses on synthesizing ideal parameters for SCI and provides recommendations on how to standardize preclinical research to improve the translational potential of PBM.

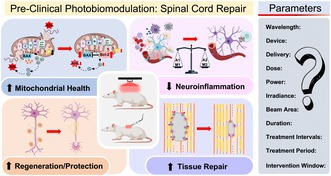

**Abbreviations**
A1: reactive astrocytes
A2: reparative astrocytes
Arg1: arginase 1
ATP: adenosine triphosphate
BBB: Basso, Beattie and Bresnahan
cAMP: cyclic adenosine monophosphate
CC: cytochrome C
CCO: cytochrome C Oxidase
CNS: central nervous system
CSPG: chondroitin sulfate proteoglycan
DNA: deoxyribonucleic acid
ETC: electron transport chain
GPx: glutathione peroxidase
H_2_O_2_
: hydrogen peroxide
HIF‐1α: Hypoxia inducible factor 1 alpha
IL: interleukin
JAK2: Janus kinase 2
LED: light‐emitting diode
M1: reactive macrophages
M2: reparative macrophages
MDA: malondialdehyde
MMP: mitochondrial membrane potential
mRNA: messenger ribonucleic acid
MtDNA: Mitochondrial DNA
mTOR: mammalian target of rapamycin
NF‐κB: nuclear factor kappa‐light‐chain‐enhancer of activated B cells
NO: nitrous oxide
PBM: photobiomodulation
PDGF: platelet derived growth factor
PGC‐1α: peroxisome proliferator‐activated receptor‐gamma coactivator‐1 alpha
R/NIR: red/near‐infrared
ROS: reactive oxygen species
SC: spinal cord
SCI: spinal cord injury
SOD: superoxide dismutase
STAT3: signal transducer and activator of transcription 3
TGF‐β: transforming growth factor beta
TNF‐α: tumor necrosis factor alpha
TRP: transient receptor potential
VEGF: vascular endothelial growth factor
WALT: World Association of Light Therapy

## INTRODUCTION

Globally, the number of people living with a spinal cord injury (SCI) is about 15 million.^1^ Of these, 59% are young adults aged between 18 and 35 years^2^ who are two to five times more likely to die prematurely compared with the general populace^3^; one in three is completely paralyzed, 80% lack bladder/bowel control, and 40% of those hospitalized are in respiratory failure.^3^ They often arise from traumatic causes such as vehicle accidents (46%) and falls,^4^ and result in a destructive neurological and pathological state^5^ characterized by impaired neural signal conduction and complete or partial loss of autonomic, sensory, and motor function.^5^, ^6^, ^7^

Following the initial injury, chronic atrophic changes occur to tracts at the lesion site^8^ and continue for up to about 1 year thereafter.^9^ Due to the ongoing degeneration of ascending and descending pathways^10^ there is also reorganization of both the somatosensory and motor cortex as a consequence of attenuated signal input,^11^ hence a need for acute intervention. The interrelated secondary mechanisms, which occur within hours and even months thereafter,^12^ are currently a major hurdle to the development of effective and affordable restorative therapies.^13^, ^14^ Often, promising strategies fail to show efficacy in clinical trials, such as pharmaceutical approaches^15^ and bioscaffolds,^16^, ^17^ or do not surpass benefits obtained with conventional physical therapy, including exoskeletons,^18^, ^19^ due to the multifaceted nature of the injury. Currently, neuromodulation is one of the leading fields, restoring motor, sensory, and autonomic pathways, and has helped spinal cord injured patients regain some functional capability.^20^ However, this approach does not address the secondary damage or inhibitory environment, which needs to be stabilized, and treatment costs may be too expensive for most patients.^21^ It is evident that we need a modulatory approach to reduce secondary injury cascades, ameliorate inflammatory responses, and enhance reparative mechanisms to encourage optimal recovery. For this reason, photobiomodulation (PBM) in the red/near‐infrared spectrum (R/NIR) is becoming a promising therapy since it restores cellular equilibrium and can promote the regenerative capabilities of the central nervous system (CNS).^22^, ^23^, ^24^ Unfortunately, ill‐defined dosimetric criteria, contradictions in the literature, and suboptimal clinical outcomes are hindering the field's progression. To ensure the complexity behind treating this injury is better understood, the initial half of the review will provide an in‐depth description of the interrelated cellular events that occur during and after SCI. Once clarified, details on how PBM can be incorporated and potential avenues for improvement will be discussed with the aim of promoting PBM as an accepted and more broadly applied therapy for human SCI.

## PATHOPHYSIOLOGY

### Vascular damage and ionic imbalance after SCI

The force of the initial insult results in extravasation of leukocytes and red blood cells into the spinal cord (SC) parenchyma,^5^, ^25^ and free radical production is elevated due to the presence of iron, which catalyzes hydrogen peroxide (H_2_O_2_).^25^ Conditions including hemorrhage, ischemia, edema, and ongoing inflammatory responses damage cytoskeletal integrity^5^, ^25^ and disrupt the selectivity of ion channels.^5^, ^25^ Extracellular glutamate levels rise following depolarization of mitochondrial membranes^26^ and expulsion of cytoplasmic contents.^27^ This neurotransmitter binds to ionotropic and metabotropic receptors, forming hyperpermeable calcium channels,^26^, ^28^ inducing an influx of calcium into neuronal, glial, and endothelial cells.^28^ Astrocytes amplify this,^28^ they swell due to elevated levels of extracellular potassium ions and respond by releasing their intracellular ions and excitatory amino acids.^29^ These bind onto neuronal receptors and further augment calcium influx.^29^ Calcium is taken up by Na^+^/Ca^2+^ exchange on the mitochondrial membrane, which activates various proteolytic enzymes,^26^ protein kinases, and phospholipases, inducing calpain‐mediated protein degradation and oxidative damage attributed to mitochondrial failure.^28^ Specifically, calcium‐activated phosphatases dephosphorylate cytochrome c (CC) and induce rapid electron transport chain (ETC) flux during reperfusion, causing hyperpolarization of the mitochondrial membrane potential (MMP) and significant bursts of reactive oxygen species (ROS).^30^ Consequently, intrinsic apoptotic death pathways are activated in neurons and oligodendrocytes.^27^ Excessive calcium levels within the mitochondrial matrix induce continuous opening of the mitochondrial permeability transition pore upon reaching a certain threshold, which results in swelling,^31^, ^32^ reduces the inner MMP, and ceases adenosine triphosphate (ATP) production.^26^ The Na^+^/K^+^ ATPase becomes inactivated, which in turn elevates intracellular sodium upon axonal depolarization, and water, which flows alongside chloride ions, further exacerbates swelling and edema.^5^, ^25^, ^27^ Lastly, elevated sodium levels enhance the activity of Na^+^/H^+^ exchangers and increase intracellular hydrogen concentration,^27^ causing acidosis.^5^, ^25^, ^27^ Due to these interrelated events, mitochondrial function is substantially impaired, associated with a significant increase in ROS.^26^

### Free radical formation and oxidative stress

**FIGURE 1:**
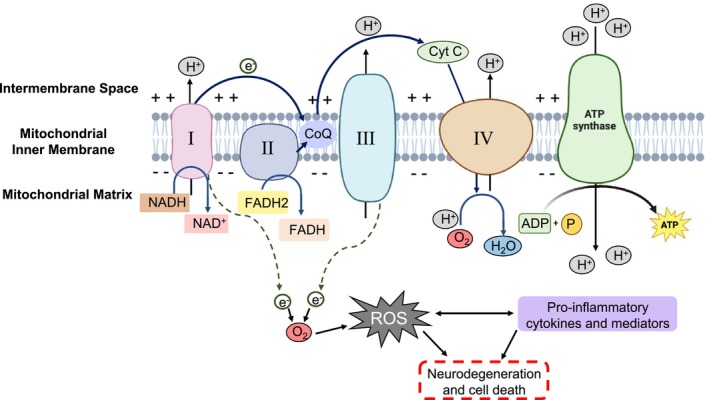
Summary of ROS production within the mitochondrial electron transport chain and subsequent damage. Figure made in part with BioRender.

**FIGURE 2:**
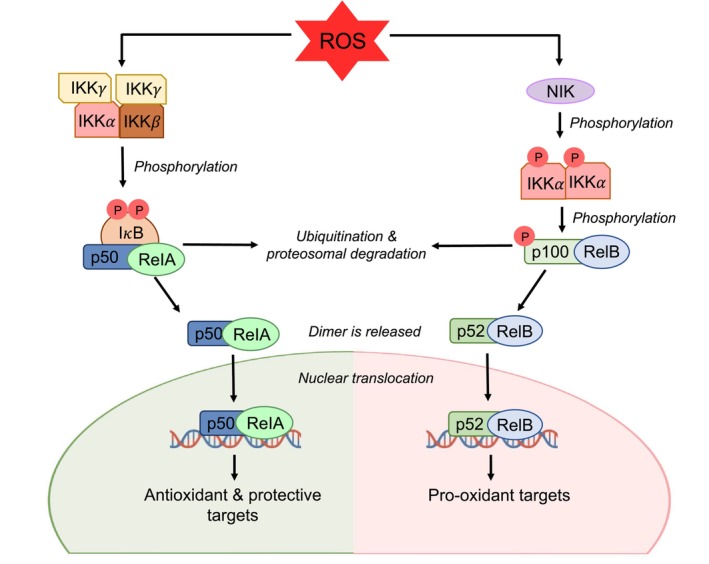
Genes transcribed following activation of the canonical (shown on the left) and noncanonical (shown on the right) pathway of NF‐κB. This highlights how ROS are key signaling molecules within the secondary cascade, and how regulating their extracellular levels can provide an escape from the cyclic inflammatory response, by upregulating the antioxidant defense system. Some antioxidant targets include manganese SOD, copper‐zinc SOD, catalase, Gpx1, and oxidant targets include inducible NOS, neuronal NOS, and cyclooxygenase‐2. Abbreviations: NIK: NF‐κB inducing kinase; IKKγ: inhibitory kappa‐B kinase subunit gamma (regulatory subunit); catalytic kinases – IKKα: inhibitory kappa‐B kinase subunit alpha, and IKKβ: inhibitory kappa‐B kinase subunit beta; IκB: inhibitory kappa‐B protein (NF‐κB inhibitor); NF‐κB transcription factors – p50: NF‐κB1 and p100/p52: NF‐κB2 form transcriptionally active heterodimeric complexes with RelA (activation inflammatory genes) and RelB (regulation of immune homeostasis); P: phosphate group. Figure modified from Morgan and Liu.^38^

Under normal circumstances, electrons are shuttled across complexes to form an electrochemical gradient; at the same time, some electrons leak from complex I (ubiquinone oxidoreductase) and III (succinate dehydrogenase) to react with oxygen and form superoxide anions (Figure 1).^26^, ^33^ Low levels of enzymatically generated ROS are necessary as they act as second messengers in several signaling pathways, are involved in the proliferation and differentiation of cells,^34^ and are required for standard redox reactions.^35^ Specifically, they can activate the redox‐sensitive transcription factor, nuclear factor kappa‐light‐chain‐enhancer of activated B cells (NF‐κB),^36^ via canonical and noncanonical pathways to either attenuate or augment oxidative stress, respectively^37^, ^38^ (Figure 2). They also promote an anti‐inflammatory and reparative environment by oxidizing cysteinyl residues in the catalytic center of protein tyrosine phosphatase 1B, thereby removing the inhibitory influence on anti‐inflammatory interleukin (IL)‐4 receptors, and prolonging cytokine expression.^39^ For this reason, abundance is regulated by antioxidant scavengers such as superoxide dismutase (SOD), catalase and glutathione peroxidase (GPx).^35^ However, under pathological conditions, ROS overwhelm these systems and saturate the enzymes.^33^, ^40^ H_2_O_2_ is generally produced at greater concentrations and selectively oxidizes methionine and cysteine protein residues.^41^ These free radicals and nitrous oxide (NO activate cytosolic poly (ADP ribose) polymerase, which attenuates NAD^+^ and induces glycolysis, leading to failure/cell death.^32^ ROS also interact with polyunsaturated fatty acids in cell membranes to produce lipid peroxyl radicals^27^ that are highly toxic and destabilize cell membranes, leading to further ionic imbalances and cellular degeneration.^28^

### Disruption of mitochondrial dynamics

Mitochondrial deoxyribonucleic acid (MtDNA) is located within the mitochondrial matrix.^26^ It is crucial for oxidative phosphorylation and ATP generation via encoding respiratory chain subunits and interacting with nuclear genes, but it is highly susceptible to oxidative damage and dysfunction.^26^ When MtDNA is mutated without *cytochrome c oxidase* (CCO), it results in respiratory chain and proton pump dysfunction, and therefore, elevated ROS^26^ (Figure 1). In turn, these free radicals bind to and damage MtDNA, further impacting respiratory enzyme synthesis and function.^26^ A homeostatic mechanism to remove damaged mitochondria is via mitophagy.^26^, ^33^ However, this is impaired by surplus ROS, strengthening the positive feedback loop of the secondary injury cascade.^33^ Under these circumstances, the outer mitochondrial membrane can rupture and expel CC, initiating mitochondrial‐induced apoptosis.^33^ When released into the cytosol, MtDNA induces a strong inflammatory response since it possesses similar properties to bacterial DNA.^33^ This, in combination with impaired mitophagy, results in increased levels of pro‐inflammatory cytokines; IL‐1β which induces mitochondrial fragmentation and ameliorates respiration, and tumor necrosis factor alpha (TNF‐α), which modifies mitochondrial structure to become condensed and hollow with reduced cristae.^33^

### Neuroinflammation

**FIGURE 3:**
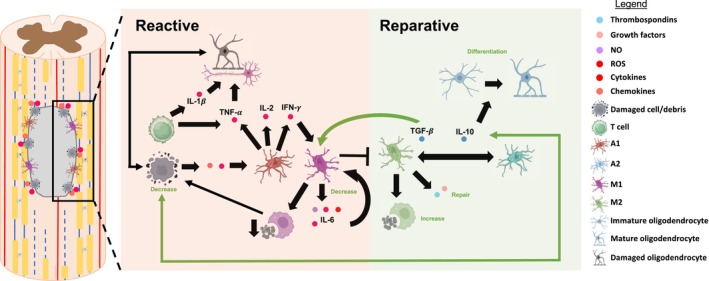
A schematic representing the cyclic relationship of neuroinflammation within the lesion site highlighting both the reactive and reparative phenotypes of glial cells. Note how inflammatory cytokines stimulated by damaged tissue reinforce glial polarization to a reactive state, resulting in further cell death, impaired debris clearance, and propagated damage. This environment becomes inhibitory to repair, leading to the formation of an impenetrable glial scar and functional loss. Image created in part with Biorender.com and Smart Servier Medical Art.

The phenotype of inflammatory cells likely exists as a continuum, with marker expression dictated by environmental cues,^42^ and cells can therefore be polarized toward a reactive or reparative state depending on circumstances (Figure 3).^27^ Neuroinflammation occurs via the release of cytokines and chemokines from damaged cells, inducing further infiltration into the lesion area and polarization into a reactive state.^25^, ^43^ Reactive T lymphocytes are destructive toward neurons and glia by releasing IL‐1β and TNF‐α.^5^ Cross‐communication with B lymphocytes results in polarization to an autoantibody phenotype, exacerbating neuroinflammation and tissue destruction.^5^ Reactive astrocytes (A1) release TNF‐α, IL‐2, and interferon gamma (IFN‐γ) to promote the reactive (pro‐inflammatory) phenotype of macrophages (M1)^27^ which in turn induces expression of additional cytokines, chemokines, NO and ROS.^44^

Elevated levels of ROS and cellular debris promote M1 macrophage polarization,^25^ which persists indefinitely and creates an inhibitory environment for reparative M2 macrophages.^45^ These M1 macrophages exhibit attenuated phagocytotic capacity, impairing tissue repair and debris clearance.^27^ They release IL‐6, which promotes further activation and infiltration of reactive macrophages and microglia.^25^ However, complete inhibition of IL‐6 can be detrimental, as this cytokine also plays important roles in axon regeneration and gliosis.^25^ Additionally, M1 macrophages release TNF‐α, which damages the blood‐spinal cord barrier and induces oligodendrocyte cell death.^25^ In contrast, reparative astrocytes (A2) release anti‐inflammatory cytokines such as transforming growth factor beta (TGF‐β) and IL‐10, which stimulate macrophages toward the M2 phenotype.^27^ These M2 macrophages express growth‐promoting factors and thrombospondins.^27^ They help alleviate inflammation through IL‐10 and TGF‐β production, while reducing activity of the NF‐κB signaling pathway, which regulates inflammation, cellular growth, and apoptosis.^27^

The anti‐inflammatory cytokine IL‐10 serves multiple beneficial functions: it stimulates astrocytes to upregulate TGF‐β, which signals microglia to downregulate pro‐inflammatory IL‐6 and IL‐1β^46^; it reduces expression of proapoptotic factors while increasing antiapoptotic factors^47^; and it enhances oligodendrocyte differentiation, which is crucial for repair following SCI.^27^

### Inhibitory environment

Following injury, there is Wallerian degeneration (anterograde) and axonal dieback (retrograde) at the lesion epicenter, accompanied by demyelination,^5^ which in humans progresses for at least 3 months,^25^ and neuronal degradation, which occurs within a year.^48^ In chronic injuries, a nonpenetrable, heterogeneous glial scar develops around the epicenter of the lesion via multifactorial cellular processes.^27^ Activated macrophages/microglia release proteolytic enzymes to increase vascular permeability, allowing further infiltration of cells.^49^ Fibroblasts deposit fibronectin, collagen, and laminin within the extracellular matrix, associated with other axon‐repulsive cues such as the semaphorins^27^ (Sema3A‐C, Sema3E, and Sema3F) and tenascins.^50^ Oligodendrocytes also transiently upregulate Sema4D and may inhibit the regenerative capacity of adult neurons via its CD27 receptor.^50^ Myelin further contributes by inducing growth cone collapse, axon retraction, and increased risk of apoptosis^25^ through Nogo,^50^, ^51^ neurite outgrowth inhibitor A, myelin‐associated, and oligodendrocyte myelin glycoprotein, binding to their co‐receptors and activating Rho/ROCK signaling pathways.^52^ Not only does this inhibitory environment prevent regeneration, but it also prevents differentiation of endogenous stem cells into neurons.^25^ Instead, it may promote the formation of astrocytes,^25^ further driving the cyclic cascade. Reactive astrocytes express inhibitory chondroitin sulfate proteoglycans (CSPGs) and upon binding to its cognate neuronal receptor, protein tyrosine phosphatase sigma, axon outgrowth is prevented by inhibiting growth cone movement.^25^ CSPG expression limits plasticity and regrowth after SCI,^53^ inhibitory effects that are amplified by interaction with the chemorepulsive family of class 3 semaphorins.^54^

Due to the imbalance in reparative phenotypes of glial and immune cells, there is a corresponding decline in extrinsic growth and neurotrophic factors, further impeding the regenerative capacity of neurons.^52^ These extrinsic signals are expressed in certain spatiotemporal gradients to promote neuronal survival, synaptogenesis, and to attract growth cones to their corresponding synaptic targets.^52^ Following SCI, there is a greater proportion of proneurotrophins (un‐cleaved derivatives of neurotrophins), resulting in opposing proapoptotic and pro‐inflammatory effects, which leads to degeneration and attenuated synaptic plasticity.^25^ There is an associated downregulation of key axonal growth pathways that are prominent during development, reduced transport of growth molecules involved in repair, and inhibition of mitochondrial transport along the damaged axons.^52^ In sum, because of the complex microenvironment imbalance,^25^ and the lack of trophic support and intrinsic growth capabilities,^20^, ^52^ the potential for long distance axonal regeneration in the mammalian SC is limited or absent.

## EXPERIMENTAL TREATMENTS

### Animal models

The choice of animal model to study SCI depends on multiple factors that include the species and strain to be used, and both the type and spinal level of injury that is made.^55^ The nature of the injury will also depend on the study's aims, as contusion and compression models are most suited toward replicating the neuropathology seen in human SCIs, whereas transection models are preferred for identifying unequivocal regenerative responses.^55^

A contusion injury is essentially achieved by applying forces to the exposed SC as a way of replicating blunt trauma^56^ and inducing each key aspect of the secondary response.^55^ The most reproducible method is dropping a narrow blunt‐ended pin to deliver a certain degree of force (kDyne) using an infinite horizon impactor (Precision Systems and Instrumentation, IH‐0400, USA) or Ohio State University device.^55^ Compression injuries constrict the cord to induce ischemic damage and are useful in identifying optimal windows for decompression.^55^ Aneurism (compression) clips are the most common technique.^55^ However, there are a lot of confounding variables that can affect injury outcomes.^57^ For instance, clips are not manufactured for multiple use and therefore force may progressively attenuate; the method of retraction can also cause further injury, and there is human error associated with how quickly and consistently clips are removed.^57^ Balloons are also used and do not damage the surrounding tissues^55^; however, reproducibility is difficult since there is no appropriate method to measure the applied force.^56^ An alternative approach is calibrated forceps, which induce bilateral compression; however, it does not represent the natural causes of injury.^56^ Transection models are achieved by partial (incomplete) or complete severing of the SC and are beneficial when exploring degeneration, regenerative therapies, neuroplasticity, or bioscaffolds.^55^

#### Species

**FIGURE 4:**
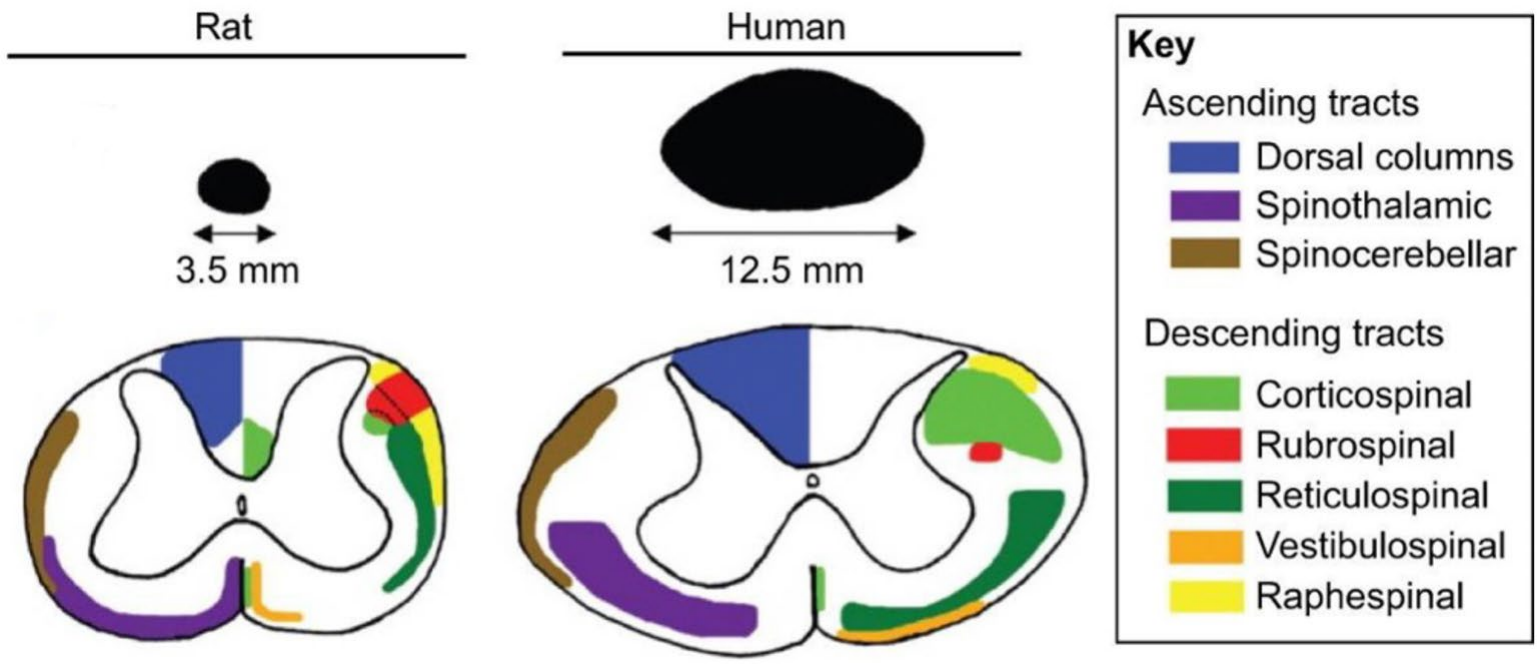
Structural comparison of a rat and human SC. Modified from Hodgetts et al.^60^

Rodents are generally preferred for preclinical studies, although mice are not commonly chosen primarily because they fail to develop a fluid‐filled cyst, typical of human SCI.^58^ Whereas rats exhibit some similar functional, electrophysiological, and morphological outcomes^55^ as they develop fluid‐filled cysts at the lesion site and fibrotic tissue, the latter due to infiltration of fibroblasts from damaged meninges.^58^ After transection, there is a loss of both motor and sensory function, resulting in permanent paralysis, and the development of an environment adverse to regeneration.^58^ Nonetheless, there are important differences regarding SC size,^58^ the inflammatory response,^59^ locomotion,^55^ and the location and function of certain descending tracts such as the corticospinal tract (Figure 4).^60^ Specifically, lesions localized to the rat corticospinal tract are not as detrimental to gait as they are for humans, and instead need to include rubrospinal and/or descending serotonergic pathways.^58^

#### Inter‐strain and sub‐strain differences

Rodents recover in a strain‐dependant manner^58^, ^61^ following SCI, linked to variability in lesion formation^61^ and immune responses,^62^ and minor differences in the location of certain tracts between strains.^58^ Discrepancies between outcomes following the same experimental model can also be attributed to genetic variability in the injury response.^63^ Through comparative studies, it has been shown that Lewis rats express a significantly greater proportion of lipid peroxidation markers, elevated circulating peripheral blood mononuclear cells (CD4 and CD8 T cells), a more vigorous and prolonged expression of activated microglia and macrophages at the lesion site, and significantly greater T lymphocyte infiltration within 72 hours following injury in comparison to Sprague Dawley and Fischer rats.^64^ The exacerbated immune response in Lewis strains is attributed to the major histocompatibility complex class II gene variant, RT1‐Db1, which results in strong affinity toward protein elements on myelin.^64^ Locomotor recovery in Lewis rats is also significantly less in comparison to Sprague Dawley and Fischer strains, which has been correlated to significantly less rubrospinal neurons.^64^ Sprague Dawley's also exhibit quicker functional recovery than Wistar and Long Evans, supposedly due to a more robust expression of genes associated with myelin structural proteins.^64^ Furthermore, injury model selection across strains influences the development of neuropathic pain and hypersensitivity.^61^

Within strains, even choosing the sex of rodents can influence functional and morphological outcomes since evidence suggests that estrogen and progesterone exert neuroprotective benefits.^65^, ^66^ Administration of the sex hormone estrogen in male rats has resulted in tissue sparing by reducing cystic cavity formation, inflammation, and myelin degradation in comparison to injured controls.^65^ It is hypothesized that estrogen improves the function of Schwann cells and confines calcium ions in the mitochondria to preserve neuronal function and prevent cell death, while progesterone augments remyelination and counteracts astrogliosis.^66^ Through this synergistic activity, there is faster functional and morphological recovery,^66^ which needs to be considered in regenerative studies since it is a confounding variable. There are even sub‐strain differences, irrespective of sex, regarding functional recovery, with reported variability in the development of mechanical hypersensitivity and size of the cystic cavity in Sprague Dawley rats.^58^, ^63^

#### Issues with transferability

Although rat preclinical experiments are helpful in assessing the potential efficacy of novel treatments, there are still issues with transferability to humans with SCI. For instance, the human SC is 3.5 times greater in diameter, and individual spinal segments are much longer (Figure 4),^60^ which is why care needs to be taken when interpreting regenerative results because what may be significant in a rodent model may not be transferable or beneficial on a larger scale.^58^ The duration of recovery in humans is generally between 6 and 12 months^58^; however, this can vary and makes determining optimal treatment windows difficult. There is also a lack of data on some aspects of human SCI, such as the exact composition of the extracellular matrix,^58^ whether other organs are affected by the initial accident,^67^ and a given subject's immune status in the days and weeks following injury, that may compromise or hinder repair potential. Furthermore, there will be genetic variation which, based on animal studies, is likely to affect SCI sequelae and treatment outcomes; such variation includes race, sex, reproductive status, age, immune status, and genetic predisposition. Considering that, after SCI, the secondary cascade is a series of interrelated cyclic events, individual treatments are insufficient to stabilize the inhibitory environment completely, and a multifaceted and modulatory approach is therefore required to restore cellular equilibrium to create a permissive environment for plasticity and regeneration.

## PHOTOBIOMODULATION (PBM) THERAPY

### What is PBM?

PBM therapy uses light within the visible and NIR range (400–1100 nm)^68^ to modulate and stimulate cellular processes^69^ via photochemical reactions to improve functional outcomes after injury.^70^ This concept was originally investigated by Endre Mester in 1967, who hypothesized that emitting photons at low power would stimulate tissue repair rather than inflict further damage.^68^ A hypothesis later verified when analyzing wound healing following tumor excision with a monochromatic 694 nm laser.^68^ Through various preclinical studies, it has been deduced that PBM efficacy is defined by the probability that a photon is absorbed by the target molecules.^44^, ^68^ This is a growing body of research encompassing numerous fields from wound healing^7^ to Parkinson's disease,^68^ due to R/NIR's ability to modulate cellular environments and restore equilibrium. PBM is a useful approach in neurological conditions such as SCI because it may prove possible to inhibit specific factors that impede neural repair while at the same time minimize alternate, off‐target effects that limit plasticity. Unfortunately, the underlying mechanisms of action are still elusive and somewhat controversial.^36^, ^68^

### Mechanisms behind PBM

Photochemical reactions upon absorption are possible since wavelengths within the 400–1100 nm electromagnetic spectrum possess energy levels (2.09–1.1 eV/photons) high enough to excite biological molecules and enhance their activity.^68^ Chromophores are sensitive to specific wavelengths as determined by their absorption spectrum.^7^, ^71^ Upon photon interaction, there is either elevated activity or conformational changes of the given chromophore, resulting in modifications to signaling molecules and transcription factor activation to improve cell viability, migration, proliferation, and/or protein synthesis.^71^ This signaling continues during the period after illumination^68^ and may explain observations of long‐term benefits.^7^, ^14^, ^23^, ^72^, ^73^, ^74^, ^75^, ^76^, ^77^, ^78^

#### Red/NIR light

The most accepted mechanism of action regarding R/NIR wavelengths is that they are absorbed by CCO^6^, ^7^, ^22^, ^34^, ^36^, ^71^, ^79^, ^80^ and any wavelengths outside of its maximum absorption spectrum (900–1100 nm) exert their benefits through transient receptor potential (TRP) channels.^33^

##### Hypothesized role of CCO

**FIGURE 5:**
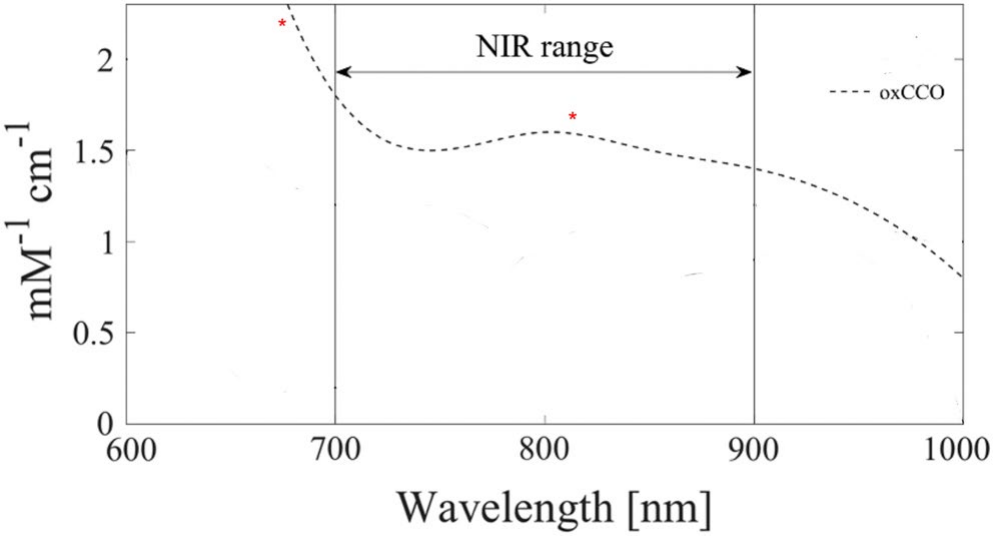
Absorption spectrum for CCO in the human body across the optical window range. Denote the range of CCO in its oxygenated form symbolized via dotted lines, with its known absorption bands at around 670 nm and 830 nm. Figure modified from Holper and Mann.^81^

CCO is the terminal enzyme in the ETC, known as complex IV, and is comprised of 13 protein subunits containing two heme: alpha and alpha‐3, two copper centers: copper A and copper B,^33^, ^71^, ^80^ and one zinc and magnesium center.^33^ Each of these subunits is involved in the electron transfer reactions^80^ and can reside in an oxidized or reduced state with differing absorption spectra.^71^ The peak absorption in red wavelengths occurs at 605 nm within the alpha heme, 620 nm for reduced copper A, and 655 nm for alpha‐3 heme and copper B.^68^ In far‐red, peak absorption is within oxidized copper B center at 680 nm; in NIR it is 760 nm within reduced copper B, and 825 nm in the oxidized state of copper A centers.^68^ Overall, the maximum absorption peaks for the given enzyme occur at 670 and 830 nm,^68^, ^81^ as denoted by Figure 5. This enzyme transfers four electrons from reduced CC to molecular oxygen, resulting in water, and simultaneously translocates four protons to drive the activity of ATP synthase.^33^, ^80^ The absorption spectrum of CCO in the R/NIR range closely parallels PBM's action spectrum^79^, ^80^, ^81^ (Figure 5), providing strong evidence for a mechanistic connection. When CCO absorbs photons at these wavelengths, its activity increases, improving mitochondrial dynamics and enhancing cellular signaling molecules.^71^

**FIGURE 6:**
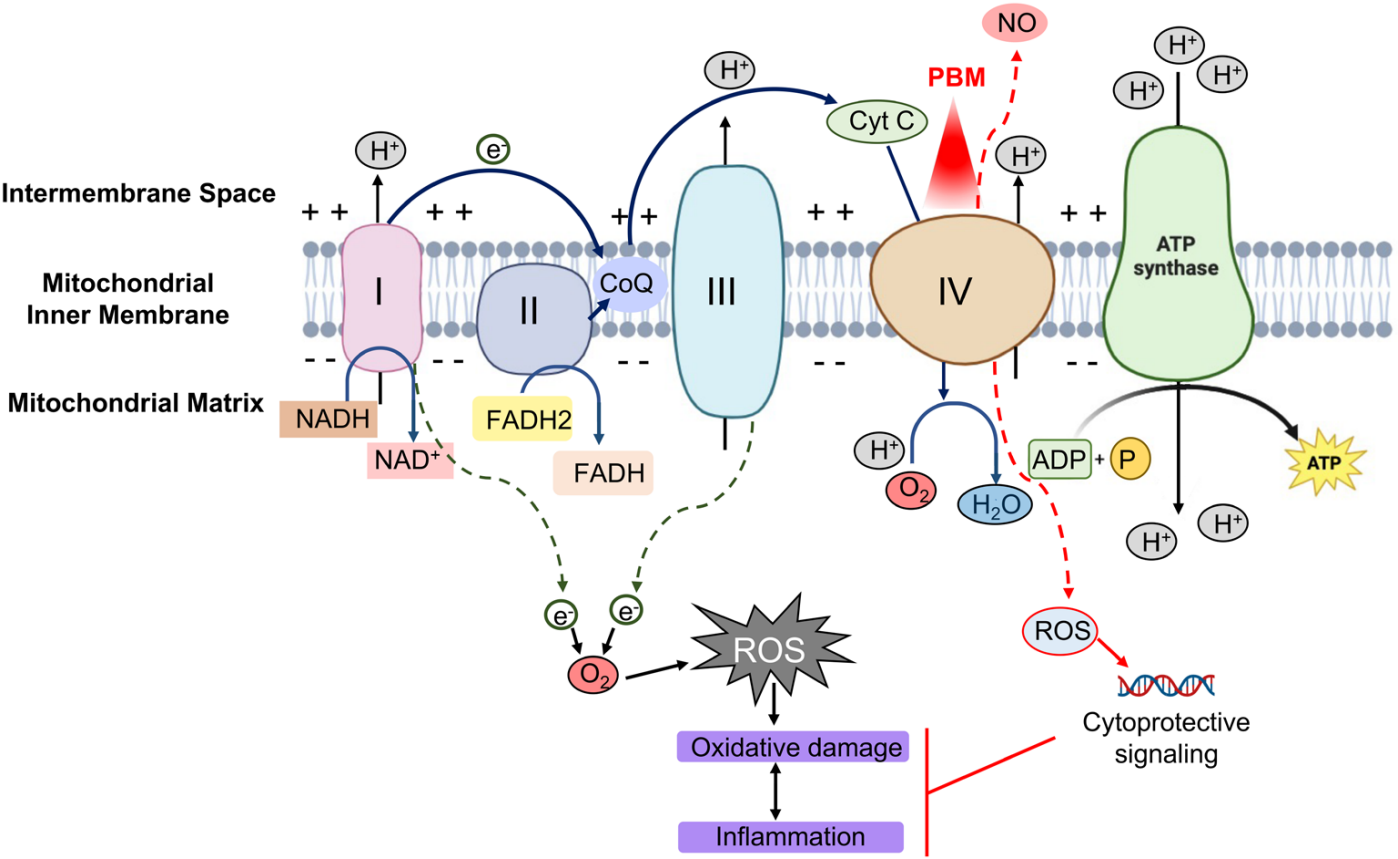
Summary of PBMs' proposed mechanism of action. Note the modulation of ROS to provide protective signaling and subsequently improved mitochondrial dynamics. Figure made in part with BioRender.

Under hypoxic or stressed conditions, CCO's activity becomes compromised by NO, which binds noncovalently to alpha‐3 heme and copper B centers.^80^ This binding competitively inhibits oxygen at a 1:10 ratio,^71^ reducing electron transport efficiency. The leading theory proposes that R/NIR light absorption can photodissociate this inhibitory NO molecule,^80^ freeing the catalytic center.^36^ This photodissociation increases the availability of electrons to reduce oxygen within the enzyme's catalytic center,^36^ ultimately improving cellular respiration.^80^ It further minimizes electron leakage and ROS production, allowing increased expression of genes regulating mitochondrial dynamics, inflammatory responses, and antioxidant activity^22^ (Figure 6).

##### Alternative hypothesis for R/NIR irradiation

While there is consensus that CCO is the central element to obtain therapeutic benefits from wavelengths between 605 and 830 nm, there is much speculation around the wavelengths exceeding this range. Some suggest cellular nanoscopic interfacial water layers may be involved, leading to changes in the physical properties of water^79^, ^82^ to improve the rotation of ATP synthase^68^, ^83^ and induce the opening of TRPV channels; influencing intracellular calcium signaling.^71^ Through selective inhibition of TRPV1 (capsazepine) and TRPC (SKF96365) calcium channels, it is evident these channels are pivotal to the action mechanism of 980 nm, but not 810 nm.^84^ Benefits obtained by wavelengths greater than 900 nm are also temperature sensitive,^84^ while some may infer this supports the interfacial water theory, it is nearly impossible to isolate its effects.

Gene knockout studies suggest there is a redundancy in the CCO components, since increased ATP and citrate levels were reported following exposure to 660 nm, despite the removal of three subunits.^85^ Irrespective of this, the enzyme still requires cellular positive feedback upon absorption of R/NIR light to exert therapeutic benefits, as when in isolation, elevated activity of the enzyme is not apparent.^86^ What is unclear, is how opposing wavelengths within its absorption spectra, 750 and 950 nm, can still provide benefits following inhibition of the given enzyme,^30^, ^68^, ^87^ and in what conditions/injuries this would be favored. Although, this is not always the case, as some studies have reported increased activity of complex III^88^ and IV (CCO)^88^, ^89^ using 950 nm^89^ and 980 nm.^88^ Since these wavelengths are believed to activate vibrational and heat‐sensitive TRP channels, they would not directly influence specific ETC complexes. It is plausible that calcium influx through the TRPV channels increases the expression of peroxisome proliferator‐activated receptor‐gamma coactivator‐1 alpha (PGC‐1α), resulting in the activation of nuclear respiratory factor 1 and mitochondrial transcription factor A to modify the expression of ETC chain complexes.^90^ Enhanced expression of PGC‐1α also negatively regulates calcium influx by reducing the efficacy of the mitochondrial uptake sites to prevent activation of intrinsic apoptotic pathways due to excessive calcium influx.^91^ Therefore, activation of TRPV1 enabling influx of calcium may result in enhanced PGC‐1α via any of three modes: (1) interaction with calcium/calmodulin‐dependent kinase to stimulate phosphorylation of cAMP response element binding protein; (2) activation of calcineurin A which proceeds to interact with myocyte enhancer factors 2C and 2D; (3) or activation of adenosine monophosphate‐activated protein kinase which directly upregulates PGC‐1α.^90^

#### Other biological chromophores

Excluding those previously mentioned, other biological chromophores may act as confounders. These include porphyrins,^92^ hemoglobin, myoglobin,^68^, ^92^ bilirubin, and lipids.^68^ Porphyrins, which synthesize heme for oxygen transport,^36^ possess a large absorption spectrum with peaks within green and red regions, and up to 250,000 nm.^36^ Hemoglobin's absorption spectra differ when it is in an oxidized or de‐oxidized state,^81^ and can have peak absorptions at 434, 556, 758, and 914 nm, whereas myoglobin can absorb wavelengths between 670 and 1000 nm.^68^ Bilirubin has a peak at 460 nm, and lipids are between 930 and 1050 nm but can absorb from 430 to 1100 nm.^68^ The presence of these needs to be considered during in vivo studies, because light emitted from the device may not reflect what is being absorbed by a particular target chromophore. This is especially important following transcutaneous delivery of R/NIR wavelengths as it means an unknown proportion of light will be absorbed by ‘off‐target’ chromophores, further complicating the ability to define what therapeutic dose is exerting favorable photobiological effects at the SC.

If delivered directly to the SC, co‐absorption by lipids may aid in enhancing the intrinsic regenerative capacity of the CNS. Following SCI, ROS produce toxic lipid peroxyl radicals after oxidizing cell membrane lipids.^27^ To minimize damage, a glycerolipid metabolic enzyme, lipin1, becomes upregulated^93^ and catalyzes phosphatidic acid; a key phospholipid^94^ which initiates the production of triglycerides to sequester lipid radicals into an inert form for storage.^93^ Unfortunately, this metabolic shift reduces the production of phospholipids such as phosphatidic acid and lysophosphatidic acid, both of which are key activators of the mammalian target of rapamycin (mTOR) and signal transducer and activator of transcription 3 (STAT3) signaling pathways for axon regeneration and repair.^94^ Upon photon absorption through CCO, PBM may indirectly help by reducing the production of ROS and subsequent lipid radicals, thereby stabilizing the environment to prevent lipin1 upregulation and maintain phospholipid metabolism. Since phospholipids comprise more than 50% of axonal membranes,^95^ direct photon absorption may increase the production of phosphatidic acid which can directly bind to and activate mTOR complex 1.^94^ Lysophosphatidic acid can also be synthesized from phosphatidic acid, where it binds to G protein‐coupled receptors and initiates signaling cascades for STAT3 activation and cytoskeletal reorganization.^94^ Sustaining phospholipid metabolism enables enzymatic production of phosphatidylcholine and phosphatidylethanolamine via Pcyt1a and Pcyt2 respectively, both of which are key lipids involved in membrane and growth cone expansion for axonal repair and regeneration.^93^ Lysophosphatidic acid is also an extracellular signaling molecule; however, it can exert dual effects depending on the given G protein‐coupled receptor.^96^ Lysophosphatidic acid has been shown to polarize M1 phenotypes and activate astrocytes by influencing their cytokine gene expression levels (Nerve growth factor, IL‐1β, IL‐4, IL‐6); however, it can also stimulate astrocytes to promote neuronal differentiation and axonal outgrowth.^96^ In sum, early intervention of PBM may promote the intrinsic reparative potential of damaged neurons through synergistic interactions with (i) CCO to improve mitochondrial health and reduce oxidative stress, and (ii) phospholipids to activate regenerative signaling pathways and membrane reparative mechanisms, while ensuring a stable extracellular level of lysophosphatidic acid to further control the inflammatory cascade.

### Secondary signaling molecules

#### ATP

ATP synthesis is increased in parallel to elevated activity of CCO^36^ and not only acts as an intracellular energy carrier to improve neuronal viability, but also acts as an extracellular signaling molecule via P2X and P2Y receptors.^97^ Purinergic signaling is mediated by purine nucleotides such as adenosine and ATP,^97^ that bind to ATP‐gated nonselective trimeric ion channels (P2X) and metabotropic G protein‐coupled receptors (P2Y).^36^, ^97^ P2X receptors are robustly expressed in neurons, glia, and nonexcitable cells (platelets, epithelial, macrophages).^98^ The receptors are involved in synaptic transmission, pain perception, inflammation, and immunomodulation.^98^ Upon binding to ATP, they undergo conformational changes enabling influx or efflux of cations (sodium, potassium, calcium) along their concentration gradient.^97^, ^98^ Binding of ATP to P2Y receptors activates stimulatory or inhibitory G proteins which influence downstream secondary signaling molecules such as cyclic adenosine monophosphate (cAMP).^97^

#### cAMP

Elevated levels of cAMP are commonly reported following PBM, partially due to the endogenous release of prostaglandin E2 by macrophages.^36^, ^99^ Prostaglandin E2 elevates cytosolic cAMP levels via G protein‐coupled receptors,^99^ which activates protein kinase A type I and results in phosphorylation of cAMP response element‐binding protein.^36^, ^99^ This protein then binds to the CRE domain on DNA to either inhibit or activate gene transcription.^36^ In addition, upon the influence of prostaglandin E2, TNF‐α transcription is inhibited to attenuate the immune response.^36^, ^99^

#### Calcium

Following R/NIR irradiation, calcium levels are restored via increased activity of Na^+^/H^+^ and Ca^2+^/Na^+^ antiporters, and calcium pumps.^100^ This is crucial for SCI repair, since calcium ions are critical intracellular messengers for neuronal survival^101^ and an array of cellular processes encompassing neuronal transmission,^102^ synaptic plasticity, regulation of ion channels, energy production,^103^ gene expression, and protein synthesis.^103^ Following glutamate‐induced excitotoxicity, elevated calcium activates nitric oxide synthase, which inhibits complex IV activity and results in overproduction of ROS from complex II and III, leading to mitochondrial dysfunction and apoptosis.^103^, ^104^ In turn, these ROS also modify redox‐sensitive calcium signaling proteins and transporters, which further augment the dysregulation of calcium ions.^104^ Poly ADP ribose polymerase 1 also becomes activated, which depletes NAD^+^, resulting in glycolysis failure and cell death.^101^ Therefore, through modulation of calcium ions to improve mitochondrial health, PBM is also stabilizing the extracellular environment and indirectly reducing neuronal and oligodendrocyte cell death, as they are highly susceptible to oxidative and metabolic distress.^27^

#### ROS and NO

**FIGURE 7:**
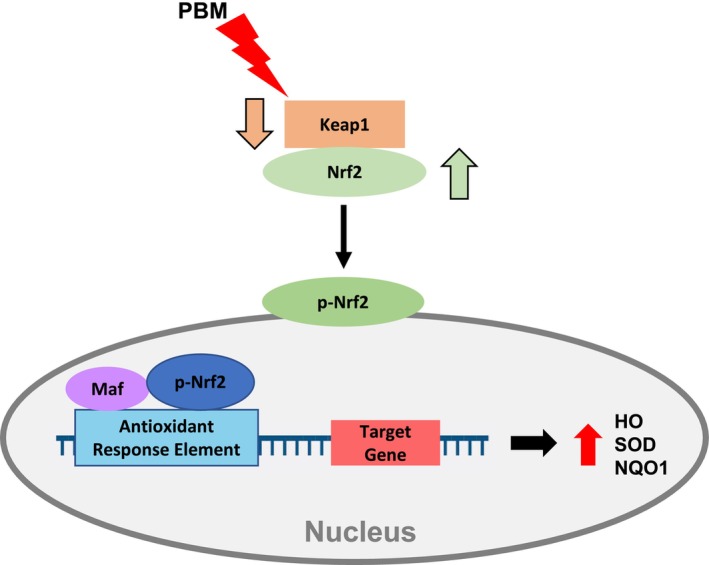
A representative diagram of one of PBMs' methods to alleviate oxidative stress via disinhibition of Nrf2 to upregulate the mitochondrial antioxidant defense system. Abbreviations: HO, heme oxygenase; Keap1, Kelch‐like ECH‐associated protein; Nrf2, nuclear factor erythroid 2‐related factor 2; p‐Nrf2, phosphorylated Nrf2; NQO1, NAD(P)H quinone oxidoreductase 1.

PBM may modulate ROS levels to exert beneficial secondary signaling via the canonical NF‐κB pathway, and therefore simultaneously downregulate oxidants and increase antioxidant synthesis^37^, ^38^ to alleviate stress and promote a homeostatic environment (Figure 2A). The redox‐sensitive transcription factor, nuclear factor erythroid 2‐related factor 2, also increases following PBM therapy^105^ and disinhibited by downregulating Kelch‐like ECH‐associated protein 1 (Keap1).^106^ This enables translocation to the nucleus, where it binds onto the antioxidant response element on corresponding promoters to increase synthesis^106^ of heme oxygenase, SOD, and NAD(P)H: quinone oxidoreductase 1^105^ (Figure 7).

NO is a versatile intra‐ and extracellular signaling molecule involved in neuronal transmission, immunomodulation, inflammatory responses, ion channels, phagocytosis, and vascular homeostasis.^107^ It is chargeless and contains an unpaired electron enabling it to act as an oxidant or reductant, and interacts with ROS to form reactive nitrogen species with associated cellular signaling effects.^107^ NO can influence genes encoding transcription factors, regulators of the cell cycle and mitochondrial oxidative phosphorylation, promote expression of inflammatory cytokines (TNF‐α, IL‐8, macrophage inflammatory protein 1‐α),^39^ and act as a free radical scavenger.^108^ It is also capable of counteracting the inflammatory cascade by reducing cellular levels of inducible NOS^108^ to alleviate inhibition of the cyclooxygenase 2 enzyme and increase production of prostaglandin E2, which as previously mentioned, ceases the synthesis of TNF‐α and promotes IL‐10.^39^ In addition, NO was noted to prevent superoxide production by inhibiting NADPH oxidase in neutrophils.^108^, ^109^ NO is reportedly increased following PBM, and may arise from elevated CC levels, a by‐product from CCs cleaved metal complexes,^37^ or upon displacement from CCO.

### Overall effects of PBM

#### Neuroinflammation

**FIGURE 8:**
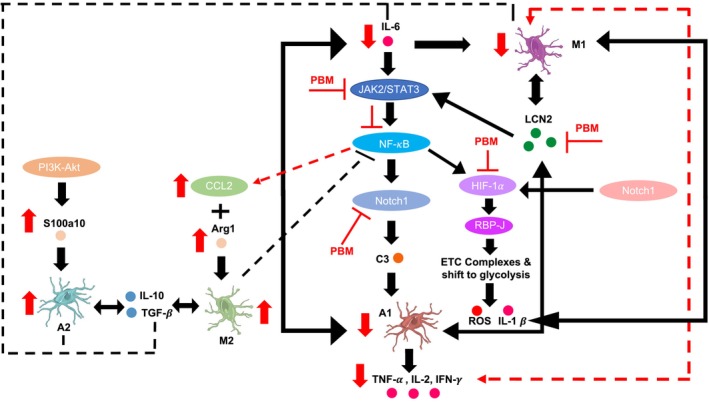
A simplified schematic diagram representing how PBM (red) influences the polarization of reactive astrocytes (A1) and macrophages (M1) to a reparative state (A2/M2). This also highlights how complicated and interrelated the signaling pathways involved are. However, it is important to note that PBM's mode of action regarding polarization is not yet completely understood. Abbreviations: CCL2, chemokine (C‐C motif) ligand 2; C3, complement 3; LCN2, lipocalin 2; PI3K‐Akt, phosphatidylinositol 3‐kinase/protein kinase B. Figure made in part with Smart Servier Medical Art.

PBM has been reported to polarize astrocytes, macrophages, and microglia to a reparative phenotype^23^, ^77^, ^110^, ^111^, ^112^, ^113^ (Figure 8), although the mechanism remains unclear. Cytokine synthesis and release is modulated^114^ through the NF‐κB pathway to downregulate IL‐1α, IL‐8,^115^ and TNF‐α, followed by an upregulation of IL‐10^70^ and TGF‐β.^115^ A recent study deduced that astrocyte polarization is dependent on activation of the Janus kinase 2(JAK2)‐STAT3 pathway, with subsequent activation of NF‐κB and Notch1 for an A1 phenotype, or phosphatidylinositol 3‐kinase/protein kinase B (PI3K‐Akt) for an A2 phenotype.^74^ However, there were discrepancies noted following SCI, as Notch1 signaling was not influenced even after R/NIR irradiation.^74^ Notch‐1‐hypoxia inducible factor 1 alpha (HIF‐1α)/NF‐κB signaling is required to induce reactive macrophages,^116^ as HIF‐1α transcribes genes involved in adaptation to hypoxic conditions,^36^ and therefore differing signaling pathways may be required for glial polarization. Binding of the Notch1 ligand to its cognate receptor results in translocation of the intracellular domain by γ‐secretase to the nucleus where it further binds to the transcription factor RBP‐J, to activate target genes.^116^ The intracellular domain also interacts with mtDNA to upregulate respiratory chain complexes and pyruvate dehydrogenase phosphate 1 for metabolic reprogramming.^116^, ^117^ When macrophages polarize to an M1 phenotype, they shift their metabolic pathway to glycolysis via Notch1 activation,^117^ which enables increased synthesis of ROS for HIF‐1α stabilization and IL‐1β expression.^118^ Activation of NF‐κB via cytokines increases transcription of HIF‐1α in macrophages and subsequent synthesis of pro‐inflammatory cytokines, further stimulating reactive polarization of other immune cells and effectively forming a cyclic cascade.^119^

R/NIR light significantly attenuates transcription and translation of HIF‐1α, Notch1, and NF‐κB following SCI resulting in a reduction of pro‐inflammatory cytokines and M1 phenotypes.^117^ NF‐κB also transcribes chemokine (cc motif) ligand‐2^120^, ^121^ which is required for polarization of macrophages and is notably elevated following PBM.^122^ Chemokine (cc motif) ligand‐2 secretion was increased following irradiation of cultured dorsal root ganglion cells after H_2_O_2_ induced stress, and when this media was administered to M1 macrophages, there was greater polarization to an M2 phenotype.^122^ There was also simultaneous attenuation of inducible NOS and TNF‐α, and increased expression of arginase 1 (Arg1).^122^ Activated astrocytes and microglia synthesize and excrete lipocalin 2, which can promote reactive polarization of other glia via autocrine signaling.^112^ R/NIR irradiation significantly inhibited synthesis of lipocalin 2, JAK2, and STAT3 resulting in reduction inflammatory cytokines and glial reactive surface molecules to promote reparative phenotypes.^112^ Lipocalin 2 may also exert an anti‐inflammatory effect by modulating NF‐κB‐STAT3 signaling.^123^

#### Neuroprotection

**FIGURE 9:**
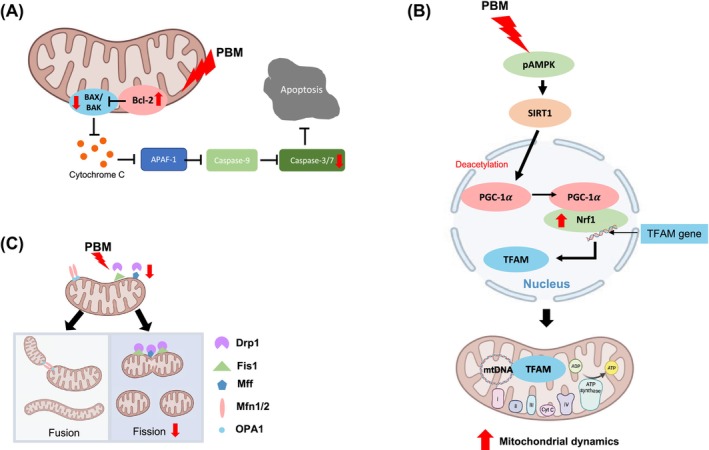
PBM exerts neuroprotective benefits via downregulation of the proapoptotic pathway (A), improving mitochondrial dynamics through modifications of mtDNA (B), and fission‐related proteins (C). Abbreviations: APAF‐1, apoptotic protease activating factor 1; BAK, Bcl‐2 antagonist killer 1; Drp1, dynamin‐related protein 1; Fis 1, fission protein 1; Mff, mitochondrial fission factor; Mfn1/2, mitofusion 1/2 proteins; Nrf1, nuclear respiratory factor 1; OPA1, optic atrophy 1 protein; pAMPK, phosphorylated adenosine monophosphate‐activated protein kinase; PGC‐1α, peroxisome proliferator‐activated receptor‐gamma coactivator‐1 alpha; SIRT1, silent information regulator 1; TFAM, mitochondrial transcription factor A. Image in part created with Biorender.com.

As previously explained, PBM is presumed to exert its neuroprotective effects by modulating ion channels, attenuating the secondary cascade, and improving mitochondrial dynamics. ETC chain complexes are upregulated,^105^ and signaling pathways associated with apoptosis are modulated. Specifically, PBM activates protein kinase B and inhibits glycogen synthase kinase‐3 beta, preventing its interaction with proapoptotic protein, B‐cell lymphoma 2 (Bcl‐2) associated X (Bax).^36^ Bax and caspase‐3^75^ are significantly reduced, while Bcl‐2 is increased,^105^, ^124^ promoting survival (Figure 9A). PBM achieves this by activating silent information regulator 1 through phosphorylation of adenosine monophosphate‐activated protein kinase, which subsequently deacetylates PGC‐1α to enable activation of nuclear respiratory factor 1.^105^ This transcription factor then proceeds to activate mitochondrial transcription factor A to modify mtDNA transcription and regulation for improved mitochondrial dynamics^105^ (Figure 9B). Inhibition results in attenuated protein levels of each complex (I–V) and ATP, as well as substantial neuronal apoptosis and impaired functional recovery.^105^ Mitochondrial dynamics are further improved via increasing the proportion of elongated (healthy) mitochondria,^33^, ^105^, ^124^ and downregulating fission‐related proteins; dynamin‐related protein 1, mitochondrial fission factor, and fission protein 1 close to control levels (Figure 9C), which further attenuates ROS production and inflammation.^33^

#### Regeneration

Due to elevated reparative phenotypes,^74^, ^77^, ^110^, ^111^, ^112^ attenuated oxidative stress,^75^, ^76^, ^124^, ^125^, ^126^ neuroprotection,^75^, ^110^, ^111^, ^124^, ^125^, ^126^, ^127^ cell proliferation and neovascularization,^128^ PBM is able to promote tissue sparing, reduce demyelination, and improve function. This stable environment may minimize myelin debris and oligodendrocyte cell death, thereby attenuating extracellular levels of myelin‐associated inhibitory proteins. In turn, fibroblasts around the lesion may decrease their production of inhibitory semaphorins and tenascins, and instead favor the release of pro‐regenerative growth factors. It is plausible that PBM could also promote the intrinsic regenerative capacity of damaged neurons by favoring phospholipid metabolism to activate mTOR/STAT3 signaling pathways.^94^ Evidence suggests that PBM can reduce CSPG expression,^14^ and influence the generation of certain growth factors such as basic fibroblast growth factor, vascular endothelial growth factor (VEGF), platelet derived growth factor (PDGF), TGF‐β, and insulin‐like growth factor 1,^115^ potentially resulting in greater plasticity and enhanced axonal regeneration.^22^

### Dosimetric parameters

Widespread clinical application of PBM is hampered by a lack of accurately defined dosimetric parameters^100^ and wide variability in experimental paradigms and animal models. Specifically, there is no consensus as to the optimal criteria for wavelength, fluence (energy density), irradiance (power density), method of light induction (light‐emitting diode (LED) or laser), mode of delivery (pulsed or continuous), and timing of treatment (session duration, treatment course, time post‐injury).^100^, ^129^ Since these parameters are interrelated, omission or inappropriate selection may impede beneficial outcomes,^36^, ^70^, ^129^ and the occasional lack of detailed protocol in publications renders studies difficult to reproduce^68^, ^130^ In SCIs alone, parameters have ranged from 589^125^ to 905 nm,^77^ 25^7^ to 175,000 mW,^44^ 0.5^14^, ^72^ to 1800 J/cm^2^,^74^ 8 s^9^ to 60‐min durations^74^, ^124^, ^126^ daily, ranging from 5 days^7^ to 4 weeks^76^ (Table 1). Choice of parameters should co‐align with the varying optical properties of a tissue and the type of injury.^68^, ^129^ The nature of the light delivery must also be considered a key factor, since implanted optic fiber cables have been shown to substantially damage myelinating tracts.^131^ It may become displaced or cause other tissue damage once animals begin to show functional improvement.^44^ Additionally, external handheld devices are limited by light attenuation, which makes it difficult to determine what dose was received at the injury site.^44^ To reliably characterize parameters for SCI, PBM methodologies need to be standardized and a more efficient mode of photon delivery needs to be investigated before more human trials are considered.

**TABLE 1:** Summary of outcomes obtained from differing parameters following a thoracic SCI in a rat model.

Group	Primary authors	Injury and rat strain	Wavelength (nm)	Power and irradiance	Beam area (cm^2^)	Fluence and energy per session	Irradiation site	Intervention time	Treatment regimen	Time of analysis (dpi)	Combined outcomes	Limitations
**(1)**		T13‐L1 Moderate aneurysm clip compression (20 g) for 90 s 14, 72, 73 Male Wistar 14, 72, 73	660 14, 72, 73 Continuous laser 14, 72, 73	100 mW^14^, ^72^, ^73^ 819 mW/cm ^2^ 14, 72, 73 ***Calculated*** *507.6 mW/cm* ^2^	0.197^14^, ^72^, ^73^	0.5 J/cm ^2^ 14, 72, 73 ***Calculated*** *2.5 J/cm* ^*2*^ *per point (22.8 J/cm* ^*2*^ *total), 0.5 J per point (4.5 J total)*		30 min after SCI 14, 72, 73	9 points (5 s/point)^14^, ^72^, ^73^		Significantly increased: BBB scores, axon density, fibroblast invasion, and CSPGs. Alleviated mechanical allodynia and thermal hyperalgesia. Significantly decreased GSK3β, AQP4, demyelination, and IL‐6 (protein and mRNA). Did not significantly reduce cavity size or increase GDNF (PBM alone). Combined treatment (chondroitinase ABC or hADSC) generally resulted in significantly greater improvements than either therapy alone.	Fluence and irradiance unclear, unsure how much power and energy reached the cord, and distance of irradiation from the injury site is not specified.^14^, ^72^
	Janzadeh *et al*., 2017^**14**^						Incision site (transcutaneous)^**14**^		Daily for 14 days ± chondroitinase ABC injection 7 dpi^**14**^	28^**14**^		
	Mojarad *et al*., 2018^**72**^						Incision site (transcutaneous)^**72**^		Daily for 1 and 2 weeks^**72**^	35^**72**^		
	Sarveazad *et al*., 2019^**73**^						5 mm away^**73**^		Daily for 14 days ± hADSC injection 7 dpi^**73**^	28^**73**^		

**(2)**	Neshasteh‐Riz *et al*., 2022^**76**^	T13‐L1 Compression (90 s, 20 g/cm^2^) Male Wistar	660 Continuous laser	100 mW 500 mW/cm^2^	0.197	13.7 J/cm^2^, 2.7 J (27 s) 22.8 J/cm^2^, 4.5 J (45 ) 45.6 J/cm^2^, 9 J (90 s) 60 J/cm^2^, 11.7 J (117 s)	1 cm from skin	30 min after SCI	9 points (3 s/point) Daily for 4 weeks 9 points (5 s/point) Daily for 2 and 4 weeks 9 points (10 s/point) Daily for 4 weeks 9 points (13 s/point) Daily for 4 weeks	35	4 weeks PBM significantly increased BBB, pain threshold and significantly improved bladder function (117 s closest to control). Antioxidants: PBM (117 s) significantly increased GPx activity and reached control levels. SOD activity did not significantly differ from control (117 s greatest increase). Oxidants: PBM decreased MDA to levels not significantly different from control (greatest decrease 117 s). 2 weeks PBM significantly greater fibroblast invasion than SCI and control groups. After 4 weeks PBM, levels were significantly decreased but still significantly higher than control (117 s greatest decrease). PBM for 27 s did not significantly differ from SCI control, and the remaining energy levels did not significantly differ between each other except for BBB scores (117 s higher).	Unclear how much power and energy reached the cord, errors in graphs.

**(3)**		Moderate bilateral compression (0.5 mm forceps, 40 s) 74, 124, 126 Male Sprague Dawley 74, 124, 126	810 74, 124, 126 Continuous laser 74, 124, 126	150 mW 74, 124, 126 500 mW/cm^2^ 74, 124, 126	0.3 74, 124, 126	1800 J/cm^2^ 74, 124, 126 540 J 74, 124, 126	Embedded laser fibers 74, 124, 126 Animals anesthetized 74, 124, 126	Immediately after 74, 124, 126	Single (3600 s)^74^, ^124^, ^126^		Significantly increased BBB, neuronal survival, A2 astrocytes, SOD, total antioxidant capacity, Nrf2, HO, and NQO1. Significantly decreased stride length, cavity area, A1 astrocytes, phosphorylated NF‐kB, JAK2, STAT3 and Akt mRNA, and NT‐3, MDA, Keap1, Nox2, ROS, 8‐OHdG levels. Cytokines: significantly decreased TNF‐α, IL‐6, IL‐1β, inducible NOS, lipocalin 2, and significantly increased TGFβ, and bFGF. Improved mitochondrial dynamics: significantly reduced swelling and fission, significantly increased the number of healthy mitochondria and upregulated all ETC complexes and ATP. No significant influence on growth factors: BDNF, NGF and GDNF.	Wang et al., refers to previous parameters^**110**^ without stating modifications in the text. Unclear how much power and energy reached the cord.^74^, ^124^, ^126^ “n” values not reported^74^, ^124^ and no report on beam area, fluence, total energy, or irradiance.^**126**^
	Wang *et al*., 2021^**74**^	T10^**74**^							Daily for 14 days^**74**^	3, 7, 14, 21^**74**^		
	Zhu *et al*., 2023^**124**^	T10^**124**^							Daily for 14 days^**124**^	14^**124**^		
	Li *et al*., 2023^**126**^	T11^**126**^							For 7 and 14 Days^**126**^	1, 7, 14^**126**^		

**(4)**			810 23, 24, 75, 106 Laser fibre 23, 24, 75, 106	150 mW 23, 24, 75, 106	0.3 23, 24, 75, 106	450 J 23, 24, 75, 106	Skin contact (transcutaneous) 23, 24, 75, 106 Animals anesthetized 23, 24, 75, 106				6% power transmitted from the skin to ventral SC (~9 mW and 90 J/cm^2^)^**23**^. Significant decrease in EMG latency, lesion volume, apoptosis, MDA, the number of glia, M1 macrophages and microglia, T‐lymphocytes, TNF‐α, IL‐1β, IL‐6, inducible NOS, MCP‐1. Significant increase in BBB score, motor and sensory coordination, restored angle of rotation and ladder beam walk time, significantly increased the number of neurons, mean axon distance travelled, number of regenerating neurons, SOD, GPx, catalase, M2 macrophages, IL‐4, IL‐13, IL‐10. No significant increase in distance travelled by the animals, foot falls, stride length or base of support. No significant influence on neutrophils, Schwann cells, lymphocytes (18 h ‐16 dpi).	Not all papers included uninjured controls or reported each dosimetric variable, instead prior papers were cited such as Byrnes et al., 2005 and Wu et al., 2009. One study reported a greater irradiance^**23**^and two reported a higher dose^23^, ^24^ despite using the same beam area, power, and treatment duration as the other papers.
	Hassan *et al*., 2021^**75**^	T9^**75**^ Significant contusion (height: 25 mm, weight: 10 g)^**75**^ Female Wistar		500 mW/cm^2^ ^**75**^		1500 J/cm^2^ ^**75**^			5 points (600 s/point)^**75**^ Daily for 14 days	56^**75**^		
	Byrnes *et al*., 2005^**23**^	T9 Hemisection (dorsal transection corticospinal tract)^**23**^ Sprague Dawley		530 mW/cm^2^ ^**23**^		1589 J/cm^2^ ^**23**^		15 min after SCI^**23**^	2997 s^**23**^ Daily for 14 days	6hr, 2, 4, 14, 16, 35, 70^**23**^		
	Wu *et al*., 2009^**24**^	T9/10^**24**^ Significant contusion (height: 25 mm, weight: 10 g) T9 Hemisection (dorsal transection corticospinal tract) Sprague Dawley				1589 J/cm^2^ ^**24**^		15 min after SCI^**24**^	2997 s^**24**^ Daily for 14 days	7, 14, 21^**24**^		
	Song *et al*., 2017^**110**^	T8^**110**^ Moderate bilateral compression (forceps, 20 s) Sprague Dawley							3000 s^**110**^ Daily for 1–14 days	1, 3, 7, 14^**110**^		

**(5)**	Svobodova *et al*., 2019^**77**^	T10 Compression Fogarty balloon (15 μL saline, 5 min) Male Wistar	808 continuous + 905 pulsed MLS Laser	1000 + 25,000 mW ***Calculated*** *333 + 8333 mW/cm* ^*2*^	3	300 J ***Calculated*** *545 J, 182 J/cm* ^*2*^ *(808 nm)*	3 cm above injury Animals anesthetized	15 min after SCI	Single (545 s) Daily for 10 days	63	Significantly increased BBB, the amount of white and grey matter sparing, and M2 macrophages. Significantly decreased hyperalgesia and reduced mRNA levels of FGF2, caspase‐3, CD86 and VEGF. No difference in area of glial scar, the number of protoplasmic astrocytes at the injury site, axonal sprouting, IBA1 expression (activated microglia), apoptotic cells and did not significantly upregulate Arg1.	Unclear if treatment area is meant to refer to beam area, and irradiance or fluence is not reported. Unsure how much power and energy reached the SC, pulsed duration is not reported and therefore unable to calculate energy from the 905 nm laser, no uninjured controls included, and “*n*” values not reported.

**(6)**	Veronez *et al*., 2017^**140**^	T9‐10 Moderate contusion (150 kDyne) Female Wistar	808 Continuous laser	30 mW 1070 mW/cm^2^	0.028	500 J/cm ^2^ ***Calculated*** *151 J/cm* ^*2*^, *4 J* 750 J/cm ^2^ ***Calculated*** *227 J/cm* ^*2*^, *6 J* 1000 J/cm ^2^ ***Calculated*** *302 J/cm* ^*2*^, *8.5 J*	Transcutaneous – direct skin contact	15 min after SCI	Single (141 s) Daily for 7 days Single (212 s) Daily for 7 days Single (282 s) Daily for 7 days	7	1000 J/cm^2^ significantly improved BBB score. 1000 J/cm^2^ significantly decreased CD68 expression and tactile sensitivity. 750 and 1000 J/cm^2^ significantly reduced lesion volume. 1000 J/cm^2^ significantly decreased number of CD68+ve cells.	Unclear how much power and energy reached the SC, reports spot size instead of beam diameter, energy not reported, no uninjured controls included, and errors in y axis units.

**(7)**	Hu *et al*., 2016^**111**^ and 2020^**127**^	T10 Moderate Hemi‐contusion right dorsal horn (height: 25–50 mm, weight: 10 g) Male Wistar	670 LED array	35 mW/cm^2^ (3.2 mW/cm^2^ at ventral SC) ***Calculated*** *262 mW*	7.5	63 J/cm^2^ (At dorsal surface of skin) ***Calculated*** *472 J*	7 mm away from skin Confined in transparent box	2 h after SCI	Single (1800 s) Daily for 7 days	1, 3, 7^**111**^ 1, 3, 5, 7^**127**^	91.1% light (57 J/cm^2^) was absorbed/dispersed by tissues between the animal's dorsal surface (skin) and ventral surface of the SC. Did not significantly decrease GFAP/IL‐1 β expression. Significantly reduced pain sensitivity, mechanical hypersensitivity (to baseline), cell death, activated monocytes and astrocytes, inducible NOS producing microglia/macrophage. Significantly increased BBB, restored sensory pathway conduction and M2 macrophages.	Not all dosimetric variables reported, uninjured controls were not included in all experiments. Unsure how much of the 91.1% of light absorbed /dispersed reached the dorsal surface of the SC.

**(8)**	On‐ong‐arj *et al*., 2018^**125**^	T10 Compression (50 g, 15 s) Male Wistar	589 GV2 laser	50 mW ***Calculated*** *26,315 mW/cm* ^*2*^	***Calculated*** *0.0019*	***Calculated*** *15,789 J/cm* ^*2*^, *30 J*	Hiatus of sacrum (transcutaneous)	15 min after SCI	Single (600 s) Delivered 15 mins, 6, 12 and 24 h post SCI and then daily for 7 days	7	Significantly improved BBB and gross motor score 7 dpi. Significantly decreased cyclooxygenase in serum and SC. No significant decrease IL‐6. Promoted antioxidants: significantly decreased MDA, significantly increased SOD and CAT but not GPx. Significantly increased neuronal survival: reduced Bax and caspase‐3, increased Bcl‐2 and BDNF neuronal expression.	No ‘n’ values specified, missing parameters, reports beam diameter instead of area, method of irradiation is unclear, unsure how much power and energy reached the cord, and issues with density units.

**(9)**	Ando *et al*., 2013^**7**^	T10 Severe contusion (height: 25 mm, weight: 10 g) Female Sprague Dawley	808 Polarized laser Perpendicular or parallel to the SC	25 mW 8 mW/cm^2^ (measured at SC)	***Calculated*** *3.14*	9.6 J/cm^2^ (Measured at SC) ***Calculated*** *30 J*	Directly to cord (repeatedly exposed SC) Animals anesthetized	Immediately after SCI	Single (1200 s) Daily for 5 days	0, 21	Both orientations of light significantly increased BBB scores, however parallel light had significantly greater BBB scores than the perpendicular orientation. Both orientations significantly reduced cavity area. Parallel orientation had 1.8‐fold greater transmittance to SC than the perpendicular orientation. No significant difference in ATP levels between groups.	Reported spot diameter instead of beam area, unclear after what treatment session ATP levels were measured (first or last). Repeated anaesthesia and surgery likely affect healing.

**(10)**	Chen *et al*., 2021^**179**^	T9 Contusion (height: 8 cm, weight: 10 g) Male Sprague Dawley	630 Continuous laser	100 mW 50 mW/cm^2^	2	***Calculated*** *60 J/cm* ^*2*^, *120 J*	Skin (4 cm above skin)		Single (1200 s) Daily for 14 days ± human umbilical cord mesenchymal stem cells	21	Combined therapy had greater improvement in BBB score, NF‐200+ve cells and IHC scores.	Unclear when stem cells were transplanted and/or when PBM began. Unsure how much power and energy reached the cord, and fluence and energy not reported.

**(11)**	Kim *et al*., 2017^**78**^	T11 Contusion (height: 12.5 mm, weight: 10 g) Male Sprague Dawley	850 Continuous laser	500 mW		0.08 J, 0.4 J, 0.8 J	30 cm above lesion Animals were handheld	5 min after SCI	Single (250 s) Daily for 21 days	7, 35	All energy variations reduced inducible NOS expression. 0.8 J had greatest significant improvement in BBB score, restored the combined behavioural score and reduced TNF*‐* αclosest to baseline levels.	Beam area, irradiance and fluence not reported, unsure how much power and energy reached the cord.

*Note*: Papers were grouped if they used the same PBM parameters (first column), and outcomes were summarized across these studies to provide a simpler understanding of what each PBM regimen can obtain, to aid in the selection for future research. Based on the information provided in these articles, we independently calculated each variable and when disparities were found, the reported value has been represented in red text, and our value has been written in black and italicized text under the “ ***Calculated*** ” heading. When multiple papers have been grouped, the variables used across every paper is written in the first row, and bellow this, any deviation has been outlined under the given author. If certain parameters were not reported, this has been represented by a grey coloured cell. Ultimately, this table highlights the complexity in deciding which PBM parameters should be further explored in terms of their favourable outcomes, since important variables are often not reported, and the reported “dose” is arbitrary since the amount of light reaching the top of the cord is often not characterized, and when it is, the percentage of light has inconsistencies across papers.

Abbreviations: 8‐OHdG, 8‐hydroxy‐2'‐deoxyguanosine; Akt, protein kinase B; AQP4, aquaporin‐4; Arg1, arginase 1; BDNF, brain‐derived neurotrophic factor; bFGF, basic fibroblast growth factor; COX, cyclooxygenase; dpi, days post‐injury; EMG, electromyography; FGF2, fibroblast growth factor 2; GDNF, glial cell line‐derived neurotrophic factor; GFAP, glial fibrillary acidic protein; GSK3β, glycogen synthase kinase 3 beta; hADSC, human adipose‐derived stem cells; HO, heme oxygenase; IHC, immunohistochemistry; Keap1, kelch‐like ECH‐associated protein 1; MCP‐1, monocyte chemoattractant protein‐1; NGF, nerve growth factor; Nox2, NADPH oxidase 2; NQO1, NAD(P)H quinone oxidoreductase 1; Nrf2, nuclear factor erythroid 2‐related factor 2; NT‐3, neurotrophin‐3.

#### Light sources

Currently, there is debate regarding which light source is more effective, despite papers illustrating that lasers and LEDs are equivalent,^71^, ^80^, ^132^, ^133^ due to the proposed benefit of laser coherence and polarized monochromatic light.^68^, ^132^ Coherent light is hypothesized to interact with small structural imperfections on organelles, and these individual waves could interfere with one another and produce “speckles.”^68^, ^134^ It is unclear whether this property enables greater subcellular interactions than LEDs^68^, ^70^; however, most researchers agree that it is not required for photobiological benefits.^68^ Polarized light is theorized to be more effective due to birefringence, which occurs when the light travels perpendicular to highly structured proteins and refracts in two directions.^70^, ^135^ Polarized light irradiated parallel to collagen fibers has been shown to accelerate healing and completely repair superficial burns after 17 days compared with perpendicular light, due to less light scatter and increased dermal penetrance.^136^ This theory also extends to the SC since it comprises highly directional cells and structures such as myelinated axons and myofibrils.^131^ Originally, it was believed that white matter had a greater scattering coefficient than gray matter^131^; however, this was later attributed to the direction of light propagation^131^, ^137^ as 635 nm perpendicular to myelinated tracts is 3.5 ± 0.3 mm^−1^ and parallel is 1.6 ± 0.2 mm^−1^ in fresh human tissue.^131^ In contrast, gray matter possesses similar scattering coefficients for both directions: perpendicular; 2.7 ± 0.3 mm^−1^, parallel; 2.6 ± 0.2 mm^−1^, suggesting that discrepancies are attributed to the structural organization of cellular components.^131^ In vivo investigations following SCI comparing these two components identified greater penetrance (1.8‐fold) utilizing the parallel orientation and significantly greater functional recovery in comparison to perpendicular.^7^ These findings further emphasize the importance of tailoring each variable of PBM to the target tissue to ensure optimal outcomes are obtained.

#### Wavelength

Photon absorption is highly dependent on the biochemical composition of the tissue, which in turn dictates the optimal wavelength for a given application.^22^, ^92^ The key determinants of absorption are the type, concentration, and distribution of chromophores within the tissue.^138^ Conversely, scattering is caused by the mismatch in the refractive indices between different tissue components, such as cell membranes, nuclei, and organelles.^138^ The size, shape, and orientation of these cellular structures influence the scattering coefficient.^138^ NIR wavelengths like 980 nm generally exhibit higher absorption and lower scattering compared with visible red light (e.g., 670 nm) or other NIR wavelengths (e.g., 810 nm).^139^ However, because 980 nm falls within water's absorption spectrum, its use can lead to significant thermal effects, which is a critical consideration for tissue safety.^22^ The NIR range between 810 and 834 nm is often preferred over 670 nm due to its superior tissue penetration. This is because 810 to 834 nm has a lower scattering coefficient and is less readily attenuated (the combined effect of both absorption and scattering), leading to greater transmittance (11.3% vs. 6.6%)^44^ and deeper penetration (>30–40 mm) through the SC.^137^

#### Beam area, power, and irradiance

Beam area (cm^2^) and irradiance parameters are both commonly omitted or mis‐reported.^70^ Modifying beam area can alter the number of photons reaching a given depth for the same light source.^68^, ^139^ Therefore, as the illuminated area increases, the fluence and irradiance decreases.^139^ This becomes problematic when papers fail to report this variable since it can contribute to discrepancies across the literature. Generally, 1–500 mW of output power is used in PBM^68^; however, recently, papers have reported benefits using up to 25 W.^77^ The amount of power distributed per unit of area (W/cm^2^) can result in thermal damage if it is too high. Some believe this is merely wavelength dependent, and suggest that the general risk of heat production for PBM therapy increases if irradiance is about 100 mW/cm^2^ at 400–500 nm, 300 mW/cm^2^ at 600–700 nm, or 750 mW/cm^2^ at 800–900 nm.^68^, ^129^ However, heat production is fundamentally tied to the conversion of photon energy into thermal energy upon chromophore absorption.^138^ While scattering events change the direction of light, they do not directly contribute to heat production. Temperature increases are instead due to the relationship between incident power and the tissue's absorption and scattering coefficients,^138^ which collectively determine the total amount of energy deposited and its spatial distribution. Therefore, the risk of thermal effects is not merely dependent on power and wavelength, but also fundamentally governed by the optical properties of the target tissue and its capability for heat dissipation.

In terms of SCI treatment, this suggests the therapeutic range for power/irradiance (assuming direct delivery) should be lower for wavelengths in the red spectrum (670 nm) as they are more readily absorbed and scattered within the SC compared with NIR wavelengths (810–834 nm).^137^, ^139^ However, this also means that red wavelengths do not penetrate as deep through the SC.^137^ This highlights a complex interplay, whereby irradiance needs to be high enough to enable light transmittance through most of the tissue, while ensuring it is below the anticipated thermal range dictated by wavelength and tissue. If this relationship has not been considered, then suboptimal outcomes in preclinical and clinical studies may be attributed to incomplete coverage of the injured tissue, and it illustrates the requirement for irradiance to be adjusted in terms of the patients' age and level of injury. In SCIs, people have utilized irradiances between 1.07^6^, ^140^ and 8.33 W/cm^2^ ^77^ at 808 nm^6^, ^77^, ^140^; however, they were transcutaneously delivered and only for brief periods between 1.5^6^ and 9 minutes.^77^ Considering skin has its own optical properties which would also differ between layers, the anticipated irradiance resulting in thermal production may be larger. This further complicates transcutaneous methods, as the photon–tissue interactions would differ for each tissue layer between the skin and dorsal surface of the SC (skin, subcutaneous tissue, muscle, bone, epidural space, meninges, neural tissue), which may further contribute to variable and suboptimal therapeutic outcomes.

#### Radiant energy, fluence, and dose

Radiant energy is a product of the variables of power and time (J = W × s),^68^ which is why each parameter should be reported to ensure reproducibility. Fluence (J/cm^2^) is the amount of radiant energy delivered per unit of area.^68^, ^70^ When light is being used therapeutically, then the dose is the amount of energy per unit area (also units of J/cm^2^) delivered to the target region; it is based on an injury's pathology, penetration depth, and the desired outcomes.^68^ In vitro studies investigating PBM benefit toward key aspects of the secondary cascade have observed improvements using fluences ranging from 0.03 J/cm^2^ ^141^ with 107. 7 J/cm^2^,^88^ and in vivo studies have found significant improvements from 0.5 J/cm^2^ ^14^, ^72^, ^73^ to 1800 J/cm^2^.^74^, ^124^, ^126^ Defining optimal fluence is difficult since what is deemed effective in vitro will not readily translate to in vivo due to target tissue geometry and differing optical properties, issues that are exacerbated when testing in larger scale humans.

#### Characterizing optimal dose

##### Biphasic dose response

The biphasic dose response, also referred to as the Arndt‐Schulz law, suggests any deviation above or below the optimal dose will result in ineffective or possibly harmful outcomes.^68^, ^70^ It is theorized that this is due to the MMP, where doses too low may be insufficient to restore homeostasis and too high may increase the MMP above baseline, driving photogenerated ROS production and cytotoxic mitochondrial signaling pathways.^129^ An inverse trend, although highly variable, has been identified between mitochondrial density and dose, suggesting ineffective studies on rich mitochondrial tissues may be due to overdosing rather than underdosing, with beneficial outcomes generally observed between 1 and 7.5 J/cm^2^.^129^ Therefore, the distribution and abundance of cellular components may be a key factor toward determining optimal dose.^129^ Contradictions lie within the hypothesized optimal range due to discrepancies among papers; however, this is most likely attributed to the omission of parameters, use of different wavelengths and cell lines, and differing experimental designs. As discussed above, evidence suggests different wavelengths have their specific mode(s) of action and therefore, even with the same dose, may exert differential effects, emphasizing the importance of tailoring PBM parameters to the given condition and associated pathophysiologies.

PBM has been noted to differentially influence cells grown in isolation or in co‐cultures.^142^, ^143^, ^144^ For example, 808 nm at 1.2 J/cm^2^ promoted the differentiation of osteoblasts in isolated cultures but had a negligible influence on osteoclasts.^142^ However, when co‐cultured, PBM significantly increased the differentiation of osteoblasts and significantly attenuated osteoclast differentiation to obtain net bone marrow production.^142^ Similarly, 660 nm at 3 J/cm^2^ promoted colony growth of carcinoma cells in co‐culture with stromal fibroblasts; however, at a higher dose of 36 J/cm^2^, the abundancy and expansion of carcinoma colonies were inhibited without influencing the viability of surrounding fibroblasts.^143^ Thus, two different cellular phenotypes exposed to the same PBM parameters were differentially influenced. Lastly, co‐cultured microglia with cortical neurons subjected to 0.2 J/cm^2^ and 30 J/cm^2^ resulted in a significant increase in neuronal population, and 4 J/cm^2^ significantly increased neurite outgrowth.^144^ However, when microglia were treated separately and then co‐cultured, negligible benefits were observed.^144^ In short, although there are dose ranges hypothesized to promote cellular benefits such as metabolic activity (3–10 J/cm^2^) and biostimulation (0.5–1 J/cm^2^),^129^ it cannot be assumed that such benefits will apply in all conditions. Thus, while numerous publications support the effectiveness of the PBM approach,^88^, ^113^, ^141^, ^145^, ^146^ uncertainty about its efficacy may be a result of variability in the characterized doses which can have stimulatory or inhibitory effects, all dependent on chosen wavelength, cell phenotype, single or mixed cultures, type and extent of stress used, and the experimental model.

##### Bunsen–Roscoe law of reciprocity

The Bunsen–Roscoe law of reciprocity states that photobiological effects are dependent on the number of absorbed photons irrespective of irradiance and treatment duration.^68^, ^129^ However, there are contradictory papers suggesting the pertinence of irradiance and rate of dose delivery.^147^, ^148^, ^149^, ^150^ This was evident among isolated mitochondria with significantly greater complex IV activity at 1 mW/cm^2^ in comparison to 667 μW/cm^2^ and 2 mW/cm^2^ at the same dose; 2.4 J/cm^2^, with a clear positive correlation between increased activity and duration of exposure.^147^

#### Irradiation time and treatment intervals

Irradiation has been delivered over prolonged periods to maintain low power levels; however, more recently, researchers have used higher power for more acute periods.^20^, ^68^, ^130^, ^132^ The repetition of treatments has also been noted to influence outcomes, where too frequent treatments can result in undesirable effects, while too few will have negligible impact.^68^ An interplay that can relate to both treatment interval and course duration. For example, daily (12 min) transcranial irradiation (810 nm, 18 J/cm^2^) to treat traumatic brain injury in mice had significantly greater cognitive outcomes when applied for three consecutive days compared with 14 consecutive days.^151^ The cognitive function was assessed using the Morris water maze test, which showed that the group receiving three treatments had a much shorter latency time and better learning capability than the group receiving 14 treatments.^151^ A phenomenon likely related to the biphasic dose response, where insufficient duration between sessions and/or continuing treatment when imbalances are no longer present may lead to overactivation of the target chromophore (CCO), followed by photogenerated ROS.^133^ Factors influencing this may depend on the density of the target chromophore, absorption characteristics of the given tissue, the rate and time course of pathophysiological changes, the timeframe for desired cellular and molecular processes, and the delivered dosage. The range that these categories fall into is difficult to deduce for SCI repair, but given the accelerated progression, intervals exceeding 24 hours may jeopardize sustained control of the microenvironment. It could also imply the need for a dynamic approach where parameters are adjusted to complement the current stage of recovery and associated imbalances.

### Current PBM therapies being investigated

PBM has been widely investigated in preclinical studies due to its modulatory effect shown to promote tissue repair,^6^, ^9^, ^68^ microcirculation,^68^ neovascularization and cellular proliferation,^9^, ^68^, ^128^ and reduce edema, pain, and inflammation.^7^, ^9^, ^68^, ^92^ Currently, PBM is being investigated as a potential therapy for stroke, traumatic brain injuries,^7^, ^74^ neurodegenerative diseases^7^ and SCIs.^152^, ^153^, ^154^, ^155^ It is clinically applied for rheumatoid arthritis,^7^ pain management, wound healing,^7^, ^156^ periodontal disease,^7^ acute ischemic stroke,^22^ Achilles tendinopathy, thyropiditis, psoriasis, arthritis, alopecia areata,^71^ facial rhytids, sychromias, acne vulgaris, androgenic alopecia, body contouring,^156^ muscle recovery,^92^, ^156^ and burns.^7^

### Clinical trials

Due to the complex secondary cascade following SCI, the timing of PBM intervention may well dictate the scope of recovery. To date, only a few clinical trials focusing on PBM therapy and SCIs have been published. Two randomized control trials published in 2018^154^ and 2020^155^ used the same treatment protocols. The PBM parameters were adapted from Byrnes et al., 2005^23^ and Holanda et al., 2016,^157^ using transdermal administration of 808 nm, 120 mW, 4.782 W/cm^2^, for 208 s reaching 983 J/cm^2^ per point (*n* = 5) above their injury with a quantum diode laser which was repeated three times a week for a month (*n* = 12).^154^, ^155^ Physiotherapy was employed as an adjunctive treatment that involved stretching, strengthening, as well as sensory and proprioceptive training.^154^, ^155^ Patients with partial quadriplegic or paraplegic SCIs between C3–L5 with an ASIA grade injury of B–D incurred less than a year previously were included in both trials; however, demographic characteristics of the subjects were not always reported. This is important because race, sex, age, immune status, and time since as well as severity of the injury can affect treatment outcomes, especially since the concentration of melanin in the skin impacts transdermal light penetration.^158^, ^159^ Electromyograph recordings of the brachial bicep (quadriplegics) and femoral quadricep (paraplegics) muscles were taken at baseline, immediately after treatment, and at 30 days.^154^ There was no significant difference between the treatment (*n* = 13) and placebo (*n* = 12) group immediately or after 30 days during rest or contraction,^154^ which suggests PBM did not augment the benefit of physiotherapy. The subsequent phase II trial used ASIA sensory and motor scales to grade functional improvement. There was a significant increase in the strength of key muscles and improved sensation to light touch and pin pricks after 30 days of treatment (*n* = 13).^155^ Despite functional improvements, there was no significant difference between groups regarding perception of their health.^155^ Since significant values were only identified following subjective measures, it is difficult to confidently infer whether this treatment regimen surpasses current therapeutic practices such as physiotherapy. Suboptimal outcomes may reflect the limitations of PBM in terms of its therapeutic potential for intermediate and/or chronic injuries once the inhibitory glial scar has formed. Wallerian degeneration (2–48 h) and cortical changes begin within hours after injury^28^, ^160^ due to reduced input from damaged SC neurons.^160^ These remodeled cortical circuits also possess weaker connectivity as they are mostly comprised of immature filopodia spines.^160^ Therefore, initiating PBM treatment and physiotherapy within 48 hours will both protect and strengthen neuronal circuits to achieve the maximum amount of tissue and functional preservation. While PBM therapy can reduce the production of inhibitory regenerative factors in acute injuries,^14^ removing them from matured, highly dense, and heterogeneous networks may be unattainable.

In 2022 a phase I clinical safety study of an implanted scattering fiber was reported.^152^ Twelve participants with an ASIA grade B SCI between T3 and T12 occurring within 24 hours were recruited and had the scattering fiber implanted directly above (1 cm distance) the cord during their decompression surgery.^152^ The optic fiber was 600 μm in diameter and emitted 810 nm for 30 min at 300 mW, 23 mW/cm^2^ was received at the injury site, for 7 days.^152^ Patients had a significantly greater number of white blood cells, neutrophils, and high‐sensitivity c reactive protein after 3 days indicating an immune response was elicited^152^; however, this is likely attributed to the decompression surgery. Minor improvements in their ASIA sensory and motor scores occurred at the 3 month follow‐up; however, no placebo controls were included, and any observed improvement may be attributed primarily to the decompression surgery.^152^

Only one published randomized control clinical trial was identified for PBM therapy on chronic SCI subjects.^161^ Twenty‐four subjects with an ASIA grade of A–D and an injury between C3–L2 which had occurred 2–136 months prior to the trial, aged between 28 and 65 (18 males, 6 females) were included in the study. Twelve uninjured control subjects (7 males, 5 females) were also included.^161^ Note that in humans, an injury is considered chronic from 6 months onwards.^162^ An elbow venous puncture was made to insert the fiber optic needle connected to a He–Ne laser illuminator. Light (7346.93 J/cm^2^ of 632.8 nm over 3600 s with a beam diameter of 0.00196 cm^2^ at 2.04 W/cm) was presented for 15 days across 3 weeks.^161^ Blood samples were extracted at baseline, 10 and 15 days after treatment initiation to isolate white blood cells and measure their mtDNA and ATP levels for mitochondrial function as well as serum for oxidative stress markers: malondialdehyde (MDA) and total antioxidant capacity.^161^ Baseline measurements revealed significantly greater levels of oxidative stress and mitochondrial dysfunction in chronic SCI patients compared with uninjured controls.^161^ Significant improvement in these areas was observed after 15 days such that PBM‐treated subjects significantly differed from sham and were qualitatively like uninjured controls. Overall, this trial found reduced oxidative stress and improved mitochondrial function in the blood of chronic SCI patients, at least in the short term.

#### Ongoing clinical trials

At the time of writing, there are two ongoing clinical trials listed on the national registry,^153^, ^163^ one of which has already published two papers^153^ as discussed above.^154^, ^155^ These studies were identified by searching “spinal cord injury” as the condition/disease with the following terms trialed separately for intervention/treatment: “photobiomodulation therapy,” “low‐level laser therapy,” “low‐level light therapy,” “red light therapy,” “near‐infrared light.” The same clinical trials were identified from a PubMed search, and there was only one paper for chronic SCIs and PBM (discussed above)^161^; however, there were 61 results when only chronic SCIs were searched. When the following terms were searched individually on the national registry, there were 1896 results for “spinal cord injury” as the condition/disease, of which two were related to “gene therapy,” 52 for “cell transplantation,” 10 for “scaffolds,” 268 for “pharmaceutical” approaches, 74 for “physiotherapy,” 108 for “robotics,” 11 for “prosthesis,” 60 for “neuromodulation,” 24 for “rTMS,” 269 for “electrical stimulation” and 84 results for “implants.” Comparatively, under the treatment/intervention filter, there are 1209 results for “photobiomodulation therapy,” 256 for “near‐infrared light,” 538 for “red light therapy,” 1078 for “low‐level laser therapy,” and 582 for “low‐level light therapy.” In summary, it does appear that PBM therapy is of increasing interest to clinicians in several fields, although not obviously so for SCI treatment. It is important to note that our analysis suggests the comparative lack of PBM in SCI studies cannot solely be attributed to the complexity of the condition, since there are currently 1896 ongoing studies exploring other therapeutic avenues.

#### Issues with clinical translation in PBM therapy

For effective translation from preclinical to clinical research, the following need to be characterized: optimal therapeutic irradiance and dose (J/cm^2^ reaching the target tissue) for the given wavelength, method of administration, duration, and therapeutic window.^164^ From the literature, it is difficult to decipher which parameters most effectively modulate key aspects of the secondary cascade due to inter‐strain and sub‐strain differences in rodent models, the interrelationship between parameters, and the added complexity of differential methods and units employed to measure the same outcome. To date, PBM clinical trials, while noting these issues, have based parameter selection on published preclinical rodent studies,^154^, ^155^ likely contributing to suboptimal improvements in functional and sensory recovery. We have therefore undertaken the following qualitative analysis on rat studies (see Table 1) to further understand the effects of PBM and to try and identify optimal parameters for use in acute and/or chronic SCI.

### Comparative analysis of PBM parameters for SCIs

#### Functional recovery

Both motor (Table 2) and sensory outcomes are compared with decipher which dosimetric variables are optimal for functional recovery. It appears there may be a biphasic dose response in relation to the treatment period, since 1 week of transcutaneous therapy resulted in negligible benefits,^72^ and 4 weeks lead to a significantly lower Basso, Beattie and Bresnahan (BBB) score compared with 2 weeks of treatment^14^, ^73^ when using 22.8 J/cm^2^ (1 cm from skin).^76^ A minimum of 2 weeks is logical since after 7 days the glial scar begins to form and completely matures 2–3weeks following injury.^5^ If treatment can confine the tissue damage during this period, then greater neuronal sparing and functional preservation may be obtained. This suggests initiating treatment within hours and up to 1 week following injury may be an optimal intervention window, with efficacy potentially declining from this point onwards once the inhibitory scar and structural changes begin to arise.

**TABLE 2:** Summary table containing approximate endpoint BBB scores of all the papers included in this qualitative analysis.

Group	Author	Injury	Timepoint Post‐Injury (weeks)	BBB Score SCI	BBB Score PBM
(1)	Mojarad et al., 2018^72^	T13‐L1 Moderate aneurysm clip compression	5	8.5	12.5 (2 weeks PBM) 9 (1 week PBM)
(1)	Janzadeh et al., 2017^14^	T13‐L1 Moderate aneurysm clip compression	4	8	11 (2 weeks PBM)
(1)	Sarveazad et al., 2019^73^	T13‐L1 Moderate aneurysm clip compression	4	9	12.5 (2 weeks PBM)
(2)	Neshastehriz et al., 2022^76^	T13‐L1 Moderate aneurysm clip compression	4	7	13 (2 weeks PBM) 12 (4 weeks PBM)
(4)	Song et al., 2017^110^	T8–T10 Moderate bilateral compression	2	9	10 (2 weeks PBM)
(4)	Hassan et al., 2021^75^	T8–T10 Significant contusion	8	9	14.5 (2 weeks PBM)
(3)	Li et al., 2023^126^	T11 Moderate bilateral compression	2	7	12 (2 weeks PBM)
(3)	Wang et al., 2021^74^	T10 Moderate bilateral compression	4	10	13.5 (2 weeks PBM)
(5)	Svobodova et al., 2019^77^	T10 Fogarty balloon compression	5–9	4	11 (10 days PBM)
(6)	Veronez et al., 2017^140^	T9–T10 Moderate contusion	1	7.5	12 (1 week PBM)
(8)	On‐ong‐arj et al., 2018^125^	T10 Compression	1	0	9.17 (1 week PBM)
(9)	Ando et al., 2013^7^	T10 Severe contusion	3	7.5	9.5 (perpendicular) 10.8 (parallel) (5 days PBM)
(10)	Chen et al., 2021^178^	T9 Contusion	3	1.5	2.4 (2 weeks PBM)
(11)	Kim et al., 2017^78^	T11 Contusion	3	12.4	11.6 (0.08 J) 14.4 (0.4 J) 15. 7 (0.8 J) (3 weeks PBM)

*Note*: Authors utilizing the same parameters (Table 1) are color‐coordinated (blue or green), and authors who modified these are a darker shade. Please note that these are approximate values because we do not have access to the raw data.

Generally, studies have augmented benefits by increasing individual treatment durations to provide a higher dose. This was evident after increasing treatment by 72 s to emit 60 J/cm^2^ instead of 22.8 J/cm^2^ daily for 4 weeks (1 cm from skin).^76^ Although BBB scores were highly similar, only the higher dose reduced mechanical hyperalgesia to a threshold not significantly different from noninjured controls, unlike studies that transcutaneously applied 22.8 J/cm^2^ with direct^72^ and nondirect (5 mm from skin)^73^ contact for 2 weeks.^72^, ^73^ Potentially, the lower dose is sufficient to preserve spared fibers, evident by similar BBB scores; however, a greater dose may be required to stabilize neurophysiological changes to prevent hypersensitivity. Similarly, when treatment sessions were increased by an additional 10 min to emit 1800 J/cm^2^ ^126^ through an embedded laser fiber, instead of 1500 J/cm^2^ transcutaneously (~90 J/cm^2^ reaching SC),^110^ a greater BBB score was obtained within the same 2‐week time point and following a more severe injury. One further study used a continuous and pulsed laser emitting one and 25 W of power (3 cm from skin)^77^; however, improvements began to plateau around 5 weeks following injury and the BBB score was less than that obtained when using lower power, higher dose and longer treatment sessions.^75^ There was also no reduction in thermal hyperalgesia.^77^ This suggests there may be a power range to obtain optimal physiological benefits, where too high may saturate chromophores, and instead, a steady stream of photons is needed to exert continual absorption and cell signaling modification. Clearly, more work is required to determine which of the large array of dosimetric variables has the most impact on outcomes and longevity of benefits.

#### Regeneration, tissue sparing and post‐injury survival

Different types of SCIs do not follow the same recovery trajectory; thus, study endpoints can influence any conclusions about the full scope of PBM's benefits and its limitations. As in many studies on tissue repair and neural regeneration^165^ longer post‐injury survival times may be needed to better determine which PBM treatment regimens are most applicable to the various forms of SCI. For instance, following 2 weeks of transcutaneous PBM therapy (810 nm 1589 J/cm^2^), a greater population of axons caudal to the lesion were identified 3 weeks following a contusive SCI,^24^ but after a dorsal hemisection, previously transected neurons showing signs of regeneration were only seen at 5 weeks.^23^ The most plausible explanation for these findings is the acute modulation of immune responses, and the differences in both tissue preservation and the number of spared or damaged fibers following contusive versus transection injuries. In another instance, three studies used the same transcutaneous PBM regimen (660 nm 22.8 J/cm^2^) and mode of injury,^14^, ^72^, ^73^ however, only one identified a significant reduction in cavity size,^72^ the latter perhaps attributed to greater injury severity from the use of aneurysm clips. In a separate report, treatment duration was increased to deliver a higher dose through an embedded laser fiber (1800 J/cm^2^ 810 nm), which required at least 1 month to obtain significant reductions in cyst area,^74^ compared with significant findings following 14 days with the original dose (1500 J/cm^2^ 810 nm) and transcutaneous delivery.^110^ The PBM‐treated lesion area at this 2‐week timepoint was similar across studies, and therefore the insignificant findings are most likely attributed to injury variability due to using an inconsistent injury model (forceps compression).

There appears to be a negative correlation between treatment duration and fibroblast invasion utilizing 22.8 J/cm^2^, as 1^72^ and 2 weeks^14^, ^72^ of transcutaneous PBM exhibited significantly greater counts around the cavity in comparison to 4 weeks, which was lower than the SCI group and closer to uninjured control values.^76^ However, care needs to be taken when interpreting results because analysis was from a hematoxylin and eosin stain, which lacks specificity since it merely identifies nuclei and cytoplasmic content.^166^ Differential results cannot be attributed to the time of analysis, since processing occurred at 4^14^ or 5^72^, ^76^ weeks post‐injury, and may instead reflect the need for continual treatment during pivotal stages of the secondary cascade. Considering irradiation for 2 or 4 weeks did not significantly modify oxidant/antioxidant enzymatic activity,^76^ greater cellular infiltration compared with injury controls following short interventions (1 and 2 weeks) may instead indicate a regression in maintaining signaling cascades related to cellular infiltration and polarization.

In compression/contusion injuries the dura matter remains intact and fibrosis is caused by fibroblastic‐like cells, specifically perivascular fibroblasts, and type A pericytes.^167^ From 3‐7 days after SCI, M2 phenotypes are upregulated and signal pericytes to detach from capillaries and migrate to the lesion core for tissue and vascular repair.^168^ This is achieved by M2 secretion of (i) PDGF subunit B which binds to the PDGFβ receptor on pericytes and enables capillary detachment, and (ii) Sphingosine‐1‐phosphate which binds to sphingosine‐1‐phosphate receptor 3 on pericytes to activate the yes‐associated protein transcription factor and initiate fibroblast transformation.^168^ Pericyte‐derived cells peak at 2 weeks, thus the microenvironment needs to be tightly controlled to prevent either an excessive fibrotic or pro‐inflammatory response, especially considering fibroblasts autocrine and paracrine regulatory mechanisms, with functional outcomes governed by extracellular cues.^169^ Pericytes also influence microglial phenotypes by secreting cytokines to promote or reduce inflammatory responses.^168^ Considering this complex interrelationship between cells, cessation of treatment during this critical window may alter the trajectory for repair. For instance, PBM therapy may have upregulated M2 phenotypes before 3 days, enabling quicker pericyte recruitment and subsequent vascular/tissue repair. However, discontinuing treatment at 1 week may have shifted the environment back to an inflammatory state, especially considering this also aligns with when M2 phenotypes are typically inhibited.^5^ Therefore, the uncontrolled microenvironment may have stimulated fibroblast proliferation and inflammatory phenotypes, explaining the significantly greater cellular infiltrates around the lesion. This may also explain the similar findings following 2 weeks of PBM (although cell counts were lower than 1 week of PBM), given that this also co‐aligns with peak pericyte‐derived phenotypes.^169^ Interestingly, a minimum of 2 weeks of therapy was needed to identify significant improvements in terms of functional recovery (BBB) and tissue sparing (cyst size and myelination),^14^, ^72^ most likely indicative of maintained balance between glial phenotypes during peak immune and cellular responses.

Considering controlled pericyte proliferation can improve repair and minimize fibrotic scarring,^170^ further supported by significantly lower cellular counts around the lesion with 4 weeks of treatment,^76^ PBM therapy should be initiated as early as possible, with daily sessions continuing for at least 3 weeks following injury. This will allow: (i) immediate stabilization of glial phenotypes to minimize secondary damage, (ii) vascular and tissue reparative mechanisms to begin before 3 days after injury via pericyte recruitment; both ensuring minimal damage and fibrosis to maximize functional and tissue preservation.

#### Modulation of the immune response

##### Cytokines

Measuring changes to pro‐inflammatory and anti‐inflammatory cytokines at the transcript and protein level is critical in evaluating immunomodulatory benefits of PBM considering their multifaceted impact on the secondary cascade. A significant downregulation of IL‐6 messenger ribonucleic acid (mRNA) 4‐5 weeks after SCI has been reported.^72^, ^73^ Since IL‐6 is a pleiotropic cytokine and involved in both augmenting inflammation and stimulating tissue repair via fibrosis and neovascularization,^171^ it is imperative that modulation is tightly regulated. Perhaps, complexities associated with attenuating IL‐6 may explain the insignificant tissue sparing within these studies.^14^, ^72^, ^73^ Transcutaneous delivery of 1500 J/cm^2^ at 810 nm significantly decreased TNF‐α mRNA at 1^110^ and 8 weeks^75^ post‐injury, however, transcripts of IL‐10, TNF‐α, and IL‐1β were unable to be restored to control levels.^75^ When treatment duration was increased by an extra 10 minutes to deliver 1800 J/cm^2^ at 810 nm through an embedded laser fiber, there was greater than (IL‐1β) or equal to (TNF‐α) 50% reduction in mRNA transcripts within 3 days of treatment^74^ which surpasses the attenuation achieved by a lower dose.^75^, ^172^ This highlights a therapeutic range of fluence for 810 nm, as the estimated power transmittance to the SC from transcutaneous delivery is 6%^23^ which equates to 9 mW and 90 J/cm^2^ (27 J), and while significant modulation of cytokines occurred, greater modulation was achieved with a 20‐fold increase in fluence irradiated directly to the SC. Whether all the light delivered to the SC is absorbed and/or converted into photochemical energy to drive cellular effects remains to be answered, but this will be a pivotal finding in defining the therapeutic dose. Although as previously outlined, this value would be unique to the given wavelength and dictated by the SCs spectral properties (absorption, scatter, transmittance). Overall, if long‐lasting benefits plateau at a certain time, irradiation may need to continue for longer to stabilize any cytokine imbalance.

##### Macrophage polarization

To maintain the secondary cascade, there needs to be a balance between reparative and reactive macrophages. Significant modification to the amount of activated microglia or macrophages was not identified 9 weeks following injury,^77^ and may imply that PBM does not stimulate the activation of immune cells, but rather influences their polarization based on environmental imbalances or requirements. In the acute period, transcutaneous delivery of 1500 J/cm^2^ (~ 90 J/cm^2^) at 810 nm^110^ reported a significant reduction in M1 phenotypes (inducible NOS/CD11b) after 3 and 7 days; however, this was not apparent following 63 J/cm^2^ of 670 nm (7 mm from skin)^111^ despite notable attenuation at 1, 3, and 7 days (ED1/CD80). Red irradiation (670 nm) also resulted in greater populations of M2 macrophages (Arg/ED1) following each timepoint, with significance noted at 1 and 3 days; a finding that did not occur until 7 days post‐injury when irradiated with 810 nm (Arg/CD11b).^110^ Significant findings after 670 nm may have been masked by the large variation within the data set, as the relative counts for M1/M2 appear quite similar, suggesting reparative mechanisms are upregulated after 24 hours, with some cellular equilibrium maintained for at least 7 days. In contrast, while 810 nm reduced the relative imbalance at 3 and 7 days,^110^ there is still a clear disproportion between phenotypes. Interestingly, M2 counts increased at 3 days and decreased by 7 days within both experimental groups (SCI >810 nm),^110^ which may reflect the pathophysiology of compression/contusion injuries. Specifically, the M2 and PDGF pericyte interaction for fibroblast transformation (3 days),^168^ followed by an M1 spike (7 days), which may promote pro‐inflammatory responses of these newly formed fibroblasts and subsequent fibrosis. The increase in M2 phenotypes at 3 days also corresponds with the onset of angiogenesis, as M2 also recruit pericytes for functional neurovascularization via VEGF.^168^ Because of this delayed response, the environment is not permissive to functional neurovascularization, and as such, A2 astrocytes and pericytes are not recruited.^173^ Instead, these pericytes are most likely transformed into fibroblast at the lesion.

Overall then, an ideal treatment would modulate M1/M2 levels, and therefore A1/A2, before 3 days. This would allow A2 astrocytes and pericytes to be recruited for functional revascularization and controlled tissue repair. Since this was not apparent following 810 nm, even after using standard t‐tests known to increase the risk of false positives with multiple comparisons, it may imply that these are not optimal parameters for the given injury and potentially may result in more favorable outcomes with 63 J/cm^2^ 670 nm. Alternatively, given that 810 nm is less readily absorbed, a higher fluence may be required to exert equivalent effects.

#### Neuroprotection

Modulation of cell death is often explored via TUNEL staining and/or analysis of mitochondrial apoptotic markers. Acute general^111^, ^124^ and neuronal cell death^126^, ^127^ was assessed via TUNEL staining following red (7 mm from skin)^111^, ^127^ and NIR (embedded fiber) irradiation.^124^, ^126^ Data suggest peak neuronal cell death occurs within 24 hours^127^ and nonneuronal at 3 days^111^ post‐injury, aligning with key events of the inflammatory cascade.^5^ Note that TUNEL was not double‐labeled with a stain such as DAPI to obtain overall cell counts^111^ and thus determine the proportion of dying cells. Transcutaneous irradiation of 63 J/cm^2^ at 670 nm significantly attenuated the number of TUNEL stained cells one and 3 days post‐injury, with no significant difference between the two timepoints.^111^, ^127^ Since cortical changes can begin within hours after injury,^160^ initiating PBM within the first 24 hours may therefore aid in minimizing irreversible motor and sensory loss. After 2 weeks of irradiation at 810 nm and 1800 J/cm^2^, PBM prevented about 13% of neuronal^124^ and 16% of nonneuronal^126^ cells from undergoing apoptosis. However, despite employing the same methodologies, there was a greater number of neuronal^124^ versus nonneuronal TUNEL‐positive cells^126^ in injury controls (~58%^124^ versus 28%^126^), which may be attributed to aneurism clips affecting injury consistency.^57^ It is important to note here that TUNEL is not necessarily a definitive method for apoptosis detection since it lacks specificity and only detects the final phase following DNA breakage,^174^ therefore, it is unclear how many cells may be undergoing earlier stages of degeneration. The time that any given cell is TUNEL‐positive may also vary depending on particular conditions and circumstances.

#### Mitochondrial dynamics

Mitochondrial dynamics and oxidative stress are pertinent factors contributing to the secondary cascade and are therefore commonly explored as a measure of PBM efficacy. Specifically, mitochondrial structure and function, ROS, and the ratio between oxidants and antioxidants. Cellular stress and apoptosis lead to elevated expression of fission‐related proteins: mitochondrial fission factor, fission protein 1, which recruit dynamin‐related protein 1 to the scission site^175^ (Figure 9C). Overexpression of mitochondrial fission factor results in diminished mitochondrial activity,^176^ and elevated levels of fission protein 1 inhibit mitochondrial fusion and can induce CC expulsion to initiate intrinsic apoptosis.^175^ A single session of 1800 J/cm^2^ at 810 nm (embedded laser fiber) significantly increased the number of healthy mitochondria and counteracted the imbalance between fission‐related proteins after 14 days of treatment, apart from mitochondrial fission factor, which was only partially restored.^126^ Attenuated dynamin‐related protein 1 expression results in a corresponding decrease of pro‐inflammatory factors and ROS,^33^ which explains the observed improvements in mitochondrial structure and function. This shows how quickly photobiological changes can occur, especially in terms of minimizing mitochondrial dysfunction – an ideal treatment characteristic for SCI repair. These parameters also significantly improved ATP levels 2 weeks after injury^124^; however, when a substantially lower power and dose was irradiated directly to the cord, negligible acute improvements were observed.^7^ Note that ATP was measured immediately following irradiation (presumably the first session),^7^ and disruptions to cellular respiration would progressively increase after the initial insult. Therefore, insignificant differences between groups (uninjured, injured, and treated)^7^ are most likely attributed to the time of processing rather than the treatment parameters. Interestingly, despite ATP being measured in the same units, baseline levels between studies varied by over 1000‐fold (0.13 nmol/mg^7^ versus 180 nmol/mg^124^). Discrepancies in homeostatic levels may be attributed to age, gender, or assay specificity.

Interestingly, it appears that a yellow laser through transcutaneous stimulation of the GV2 acupuncture point is capable of modulating oxidative stress by restoring MDA levels to baseline and significantly increasing SOD, catalase, and GPx activity following 7 days of irradiation.^125^ Contrastingly, 660 nm of 60 J/cm^2^ (1 cm from skin) for 4 weeks restored balance between oxidant and antioxidant activity,^76^ however; a milder compression injury was induced. From these two studies, it cannot be deciphered whether yellow or red wavelengths may be more effective at modulating oxidative stress since measurements were not taken at the same time point post‐injury, and differing units of activity were employed. Considering lipids can absorb wavelengths between 430 and 1100 nm,^68^ it is possible that phospholipids in the inner and outer mitochondrial membrane are also capable of absorbing photons in the yellow part of the spectrum to improve mitochondrial dynamics indirectly. These lipids can directly interact with and modify complexes I–V activity, are required for effective electron transfer, and may influence the MMP by altering the volume in the inner mitochondrial space through cristae structural changes.^177^ Therefore, photon absorption may minimize electron leakage and improve the MMP to attenuate ROS production and indirectly lead to greater antioxidant activity and mitochondrial health.

#### Combined therapies

PBM outcomes are augmented when complemented with other therapies aimed at promoting the regenerative capacity of the CNS and/or controlling the secondary cascade. Greater functional improvement, tissue, and axonal sparing have been identified when combined with either human adipose^73^ or mesenchymal stem cell grafts.^178^ It is difficult to decipher whether improved benefits are from stem cells further modulating the inflammatory response or due to PBM inducing differentiation without tracking their progression via fluorophores, dividing, or differentiating markers. PBM therapy can shift stem cell metabolism to oxidative phosphorylation, forcing them to leave their niche and differentiate^80^ which may explain the greater axon count and reduced cavity area. Potentially for optimum outcomes, stem cell transplantation should occur around 3 weeks post‐injury after the peak immune response^43^ so that they are more inclined to differentiate based on reduced immunoreactive and supportive environmental cues. However, it seems that the adjunct therapy may induce optimal benefits if it targets different aspects to PBM for a multidisciplinary approach. While using the same parameters,^14^, ^73^ administration of chondroitinase ABC, which digests chondroitin sulfate glycosaminoglycans,^14^ resulted in substantially greater attenuation of regenerative inhibitors and cavity area, with greater axon preservation around the lesion site than utilizing human adipose derived stem cells.^73^ In this instance, chondroitinase ABC minimizes the inhibitory cues to regeneration at the lesion site, while PBM modulates the secondary cascade to produce a more permissive environment for neural plasticity.

## SUMMARY AND CONCLUSION

In summary, PBM therapy is an emerging field that holds potential for SCI repair due to its modulatory effects on key aspects of the secondary cascade. But as emphasized here, comparisons between experimental studies are difficult due to the use of different injury models, insufficient information on PBM parameters, and sometimes a lack of controls. Other variables include the nature of the emitting device, and the power density and dose used relative to distance from the injury site. Considering each wavelength has its own transmittance, absorbance, and scattering properties throughout tissue, this variable must be considered when drawing comparisons. Changes in post‐injury tissue reactivity, not only within but also surrounding the injury site, may also need to be considered. As outlined in Table 1, synthesizing what combination of parameters has the most therapeutic potential is highly complex and somewhat unattainable, since the reported “dose” value is arbitrary if the amount of light being transmitted from the dorsal to the ventral surface of the SC is unknown. Ideally, the percentage of photons and power absorbed by the injury site should be measured over time so that dosing can be adjusted correspondingly. To ensure reproducibility and reliability, measurement guidelines should be established by the World Association of Light Therapy (WALT) in addition to their PBM reporting framework for dosimetric parameters.^179^

In short, it is challenging to decipher optimal PBM parameters because: (1) aspects of the secondary cascade vary depending on the type and severity of injury, onset of treatment, and timing of analysis; (2) there are differential basal levels measured in naïve intact controls; (3) rodents recover in a strain and gender‐dependent manner; (4) there are varying units of measurement employed throughout the literature for the same reported outcomes, and (5) parameters are noncomparable since the amount of power and dose absorbed by the SC is unknown, may vary over time, and are unique to the delivery method and/or wavelength. Despite these many caveats, based upon comparative behavioral, morphological, and biochemical analysis as outlined above and in Table 1, the following parameters applied transcutaneously (i) 15,789 J/cm^2^ (30 J) of 589 nm,^125^ (ii) 60 J/cm^2^ (11.7 J) of 660 nm^76^/63 J/cm^2^ (472 J) of 670 nm,^111^, ^127^ (iii) 1500 J/cm^2^ (450 J) of 810 nm (~90 J/cm^2^, 27 J),^23^, ^24^, ^75^, ^110^ and directly to the cord (iv) 1800 J/cm^2^ (540 J) of 810 nm^74^, ^124^, ^126^ (Table 1) appear to result in more consistent and corroborative outcomes than the other protocols in this comparative analysis. Although delivery systems do not directly affect the therapeutic properties of light, we should still focus more on implantable methods. Such an approach will ameliorate concerns about the proportion of fluence/irradiance reaching the SC, reduce stress inflicted on animals by removing the need for repeated anesthesia and physical restraint during treatment. In terms of clinical translation, implantable methods will accelerate this process, as individual parameter optimization to account for heterogeneity in transdermal light penetration (melanin) will not be required. Given that peak cellular infiltration occurs within 1–2 weeks, and maturation of the glial scar within 2–3 weeks,^5^ we recommend initiating PBM therapy within 1–2 days (latest 1 week) and treating daily for at least 3 weeks to minimize irreversible tissue loss and repulsive regenerative signals around the lesion core.

### Future directions

WALT defines treatment guidelines for PBM for carpal tunnel and arthritis conditions,^180^ tendinopathies,^181^ and anti‐inflammatory applications,^182^ however, none are available for SCIs. To progress in this field, we need to define and reach a consensus on the most effective mode of delivery, therapeutic window, and dosimetric parameters. However, for this to occur effectively, it needs to be tailored to a specific type (compression, contusion, laceration) and stage (acute, subacute, chronic) of injury since these factors influence the secondary cascade events. For an effective standardized comparison across studies and research groups, there also needs to be at least two universally accepted standardized methodologies implemented into each project, and one for measuring the proportion of photons reaching the SC. We recommend an initial prioritization of inbred rat strains, specifically Fischer's, considering the noted major histocompatibility complex class II genetic variant found in Lewy rats (heightened immune response),^64^ and the reported effect of sub‐strain differences on research outcomes within outbred strains (Sprague Dawley, Wistar, Long Evans). While each type of SCI is important, we further recommend an initial focus on contusion models (moderate and severe) using the Infinite Horizon impactor device, unless more consistent and reproducible methods are employed for compression injuries. Minimizing variability in terms of animal selection and injury severity will further improve the reproducibility of preclinical research, help isolate technical and therapeutic differences between the complex interplay of PBM parameters, provide a standardized platform for comparisons, and also a foundation to ease universal cooperation so that collectively there is better understanding and characterization of therapeutic parameters for SCIs. Once achieved, applying parameters with the most promise to a variety of injury models will help elucidate the range of benefit or limitations that they may have and ensure optimization of parameters before proceeding to clinical trials.

## Data Availability

Data sharing not applicable to this article as no datasets were generated or analysed during the current study.
